# Applying traditional and machine learning-based GWAS approaches for marker-trait identification in wheat

**DOI:** 10.3389/fpls.2025.1734247

**Published:** 2026-01-28

**Authors:** Joel Joshua Milek, Sebastian Michel, Alexander Buchelt, Andreas Holzinger, Eva Maria Molin

**Affiliations:** 1 Unit Bioresources, Center for Health & Bioresources, AIT Austrian Institute of Technology, Tulln, Austria; 2 Human-Centered AI Lab, Institute of Forest Engineering, Department of Ecosystem Management, Climate and Biodiversity, BOKU University, Vienna, Austria; 3 Department of Agrobiotechnology, IFA-Tulln, University of Natural Resources and Life Sciences Vienna, Tulln, Austria

**Keywords:** artificial intelligence, GWAS, plant height, thousand kernel weight, tool comparison, *Triticum aestivum*

## Abstract

**Introduction:**

Complex traits arise from polygenic and interactive genomic architectures that are difficult to resolve using traditional genome-wide association study (GWAS) approaches. Machine learning (ML) provides complementary methods capable of capturing non-linear effects, improving signal detection, and enhancing predictive accuracy of marker trait associations (MTAs).

**Methods:**

Using a publicly available winter wheat dataset (CIMMYT), we evaluated several widely used traditional GWAS tools, including GAPIT, GCTA, GEMMA, sommer, and TASSEL, with respect to computational efficiency, model performance, and the consistency of detected associations. In parallel, ML approaches, such as Elastic Net, Extreme Gradient Boosting (XGBoost), Random Forest, and the hybrid TSLRF model, were assessed based on feature importance metrics and functional annotation of selected markers.

**Results:**

Despite a shared reliance on mixed linear models, the traditional GWAS tools exhibited differences in runtime and showed modest but meaningful variability in the number and overlap of MTAs. ML models recovered several associations detected by traditional methods and additionally identified novel markers, potentially reflecting non-linear or epistatic effects.

**Discussion:**

Our findings demonstrate that ML can effectively complement traditional GWAS approaches for marker-trait identification in wheat. By extending beyond additive effects, ML broadens the scope of detectable genetic signals, providing a practical way to analyze complex traits and support informed marker-assisted breeding strategies.

## 1 Introduction

Genome-wide association studies (GWAS) have significantly advanced plant genetics and breeding by enabling the identification of genetic variants associated with complex agronomic traits, such as yield, stress tolerance, and disease resistance. With the rise of high-throughput genotyping and the availability of reference genomes, GWAS became a widely adopted approach by the 2010s, particularly in major crops, such as wheat (Saini et al., 2022; Sehgal and Dreisigacker, 2022). Its application has accelerated marker-assisted selection (MAS) and facilitated the integration of genomic data into modern wheat breeding programs (Korte and Farlow, 2013). As one of the world’s most widely grown cereals and a staple food for over one-third of the global population, wheat is a key component of global food security. However, wheat is particularly vulnerable to abiotic stresses such as drought and heat during critical developmental stages like flowering and grain filling, and is also severely affected by a range of pests and diseases (Figueroa et al., 2018; Senapati et al., 2021). These biotic and abiotic stressors are projected to intensify in frequency and severity under future climate change scenarios, posing a significant threat to yield stability affecting global wheat production. Thus, MAS, which relies on genetic markers derived from GWAS, remains fundamental to breeding programs aiming to improve stress resilience and ensure yield stability in adverse environmental conditions (Chang-Brahim et al., 2024).

Traditionally, GWAS rely on statistical models to detect associations between genetic markers and phenotypic traits while correcting for confounding effects such as population structure and relatedness. Early approaches used general linear models (GLMs), which are computationally simple but prone to false positives due to population stratification (Yu et al., 2006; Price et al., 2006). Mixed linear models (MLMs) addressed this limitation by incorporating fixed effects for population structure (Q) and random effects for kinship (K), becoming a widely adopted standard despite their high computational cost for large datasets (Yu et al., 2006; Wang and Zhang, 2021). Efficiency improvements led to compressed MLMs (CMLMs), which estimate kinship at the group level (Zhang et al., 2010), and to the SUPER method, which computes kinship using a subset of trait-associated markers to enhance power (Wang et al., 2014). Single-locus models, however, often fail to capture the polygenic architecture of complex traits (Segura et al., 2012). Multi-locus approaches such as MLMM and FarmCPU improve detection power by iteratively including significant markers, although FarmCPU remains computationally demanding for large populations (Segura et al., 2012; Liu et al., 2016). BLINK further improves efficiency by replacing kinship-based random effects with a Bayesian Information Criterion–based fixed-effect framework, achieving higher power and speed without assuming uniform genomic distribution of causal variants (Huang et al., 2019). Simulation studies consistently rank BLINK among the most powerful GWAS models (GAPIT manual). Despite these advances, traditional GWAS models assume linear, additive effects and struggle with high-dimensional, collinear genomic data, limiting their ability to capture dominance, epistasis, and non-linear interactions and contributing to the problem of missing heritability (Witte, 2010; Korte and Farlow, 2013; Tam et al., 2019; López-Cortegano and Caballero, 2019; Enoma et al., 2022).

Within artificial intelligence (AI), machine learning (ML) offers promising solutions to these challenges. Unlike traditional GWAS models, algorithms such as Random Forest (RF) and XGBoost (XGB) use ensembles of decision trees to automatically capture non-linear marker effects and complex epistatic interactions, while providing robust feature-importance rankings and handling high-dimensional marker data without strict parametric assumptions (Guo et al., 2021; Elgart et al., 2022). Penalized-regression approaches like GLMnet, that apply elastic-net regularization to perform simultaneous feature selection and coefficient shrinkage, can mitigate overfitting and multicollinearity by efficiently identifying informative SNPs in large GWAS datasets (Cho et al., 2009; Bühlmann and Van De Geer, 2011). Especially in human genetics, ML-based and, more recently, deep learning-based GWAS approaches have been employed for epistasis detection, SNP marker prioritization, and SNP discovery (Arloth et al., 2020; Ghose et al., 2024; Mieth et al., 2016, 2021). Coming back to plant genetics, while ML‐based genomic prediction has seen widespread success in crop improvement (Grinberg et al., 2020; Yan et al., 2021; Gill et al., 2022; Sirsat et al., 2022; Montesinos-López et al., 2024), ML‐mediated GWAS remains relatively underexplored. For example, Yoosefzadeh-Najafabadi et al. (2022) applied Support Vector Machines (SVM) and RF to dissect yield and yield‐component traits in soybean, successfully uncovering quantitative trait loci (QTL) that co‐localized with those reported in previous studies. Liu et al. (2019) extended this work by using deep learning to predict phenotypes and assess genotypic contributions in soybean, demonstrating that their approach efficiently identifies significant SNPs from GWAS data. For wheat, recent studies have used ML approaches to extract top-ranking SNPs (Song et al., 2025; Wang et al., 2025). However, at the time of writing, only one study presented a ML-based GWAS approach, including empirical cutoff, to detect genetic regions associated with cuticular wax ester biosynthesis and early maturity (Tekeu et al., 2023). These examples demonstrate that ML has the potential to complement or even enhance traditional GWAS by leveraging the capabilities of ML algorithms, revealing previously undetected genetic associations and advancing our understanding of complex traits, thereby accelerating plant breeding.

Despite the growing number of tools and methods available, ranging from traditional ML algorithms to advanced deep learning models, systematic comparisons of their performance, robustness, and biological relevance in plant GWAS are still scarce. An early study evaluated a range of ML algorithms on human genetic data, demonstrating their ability to capture complex genetic architectures (Szymczak et al., 2009). More recently, Gill et al. (2022) compared tree classifiers (XGB, RF) with deep learning methods on plant data, revealing that traditional ML algorithms can outperform deep learning. Notably, they observed that XGB and traditional GWAS identified overlapping genomic regions for soybean traits, underscoring the potential of ML to complement traditional approaches. Yet, comprehensive evaluations that assess the strengths and limitations of these methods especially in the context of crop genomics across different plant species, traits, and genomic architectures are lacking. This gap hinders the broader adoption of ML-based GWAS in plant breeding and limits the ability to draw generalizable conclusions about their utility.

This study therefore aims to bridge this gap by providing a systematic, multi-metric comparison of selected traditional GWAS tools and ML algorithms. Our selection encompasses widely used GWAS tools (e.g., GAPIT, sommer, GCTA, GEMMA, and TASSEL) implementing linear models, which we compared to established ML approaches (such as EN, RF, SVM, and XGB) that have already found application in GWAS. For this comparative analysis, we focused on the two traits plant height (PH) and thousand kernel weight (TKW), two key traits in wheat breeding. Unlike prior comparisons, we evaluate not only computational efficiency and MTA detection but also biological relevance, model interpretability, and scalability across a real-world wheat dataset (CIMMYT; Juliana et al., 2019). By linking associated genes with functional gene annotation, we seek to evaluate how ML approaches can enrich conventional GWAS pipelines, reveal additional candidate loci, and provide interpretable metrics of marker relevance. Our central hypothesis is that ML models, when properly tuned and combined with robust feature-selection approaches, can (i) detect MTAs overlooked by conventional GWAS, (ii) offer stable, biologically meaningful estimates of marker relevance, and (iii) enhance the interpretability and resolution of trait-gene relationships. Ultimately, this application-driven comparative analysis aims to illustrate the potential of ML to enhance trait dissection and to support breeding strategies for complex traits in wheat.

## 2 Materials and methods

### 2.1 Data and preprocessing

**Figure 1:**
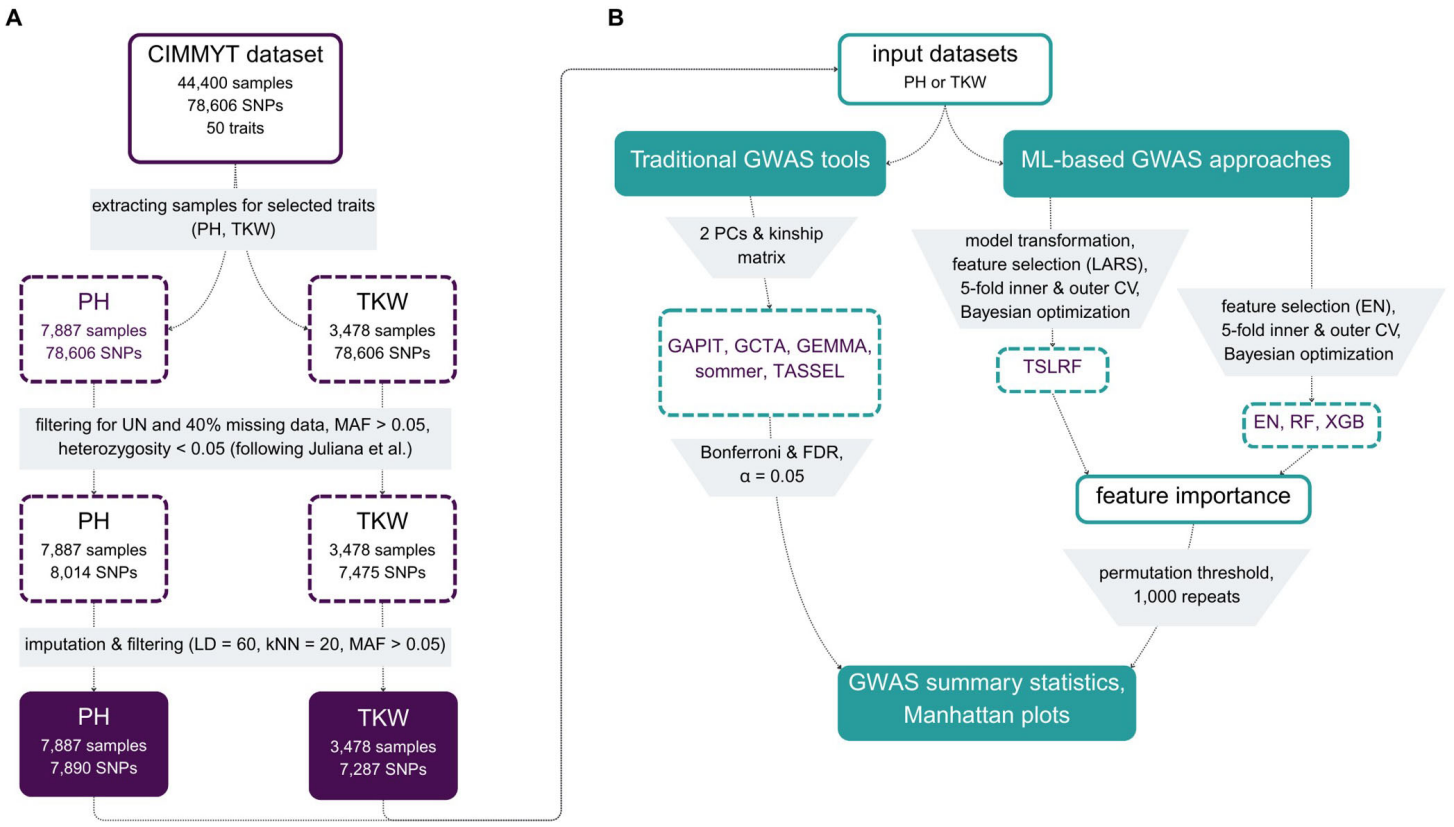
Methodological overview of data preprocessing and filtering starting from 78,148 SNPs and 44,400 individual samples **(A)** and the herein used traditional GWAS tools and ML-based approaches including used thresholds **(B)**.

To conduct our comparative study, we used a large dataset widely recognized in the field of crop genetics, published by Juliana et al. (2019). This dataset, provided by CIMMYT (International Maize and Wheat Improvement Center, Mexico), includes a total of 44,400 wheat lines for which 78,606 SNPs were identified through genotyping-by-sequencing (GBS) and 50 phenotypic traits were evaluated. After thoroughly examining the CIMMYT dataset, we created two subsets for our analysis (**Figure 1A**): the first subset was selected for the trait plant height (PH) including 7,887 individuals evaluated in first-year yield trials conducted in Ciudad Obregón, Mexico, during the 2014–2015 season, with two replicates, resembling Juliana et al.’s panel 4. The second subset targeted the trait thousand kernel weight (TKW) and comprised 3,478 individuals derived from multiple trials evaluated in Ciudad Obregón, Mexico: 766 lines from the elite yield trial (EYT) 2013-2014, 775 lines from EYT 2014-2015, 964 lines from EYT 2015-2016, and 980 lines from EYT 2016-2017, resembling Juliana et al.’s panel 1 (but seven individuals less due to removal of individuals with missing values in the quality traits). For more information on the yield trials be referred to Guzman et al. (2016) and Juliana et al. (2019). These datasets were subsequently filtered using TASSEL.v5 (Bradbury et al., 2007) as described in Juliana et al. (2019): first, a filtering of unaligned markers (UN) was done resulting in a final set of 78,148 markers (cf. Figure 1 of Juliana et al.), followed by a second filtering step for markers with less than 40% missing data, a MAF > 0.05, and less than 5% heterozygosity. Missing markers were imputed using the LinkImpute (Money et al., 2015) module within TASSEL.v5, with the following settings: Linkage Disequilibrium (LD) was set to 60, and k-Nearest Neighbors (kNN) to 20. Using the tool´s internal masking procedure to assess accuracy, the imputation error was estimated at 8% (PH) and 9% (TKW), respectively. After the imputation step, SNPs were again filtered for minor allele frequency (MAF > 0.05), resulting in the final datasets as follows: the PH dataset included 7,890 markers and 7,886 individuals, and the TKW dataset comprised 7,287 markers and 3,478 individuals. In our study, the resulting final number of markers differed from those reported by Juliana et al. (2019), who identified for panel 1 6,355 markers. The number of markers of panel 4 is not explicitly stated but falls in the range between 9,171 and 9,704 markers. In addition to this ambiguity regarding panel 4, especially the lack of information on thresholds, settings, and imputation accuracy made it impossible to replicate the filtering procedures. Furthermore, additional factors contributing to this discrepancy may be attributed to differences in TASSEL software versions. A note to the file formats: the initial dataset was in HapMap format, however, in order to use the data for Gemma and GCTA, it was converted using PLINK 2.0 (Chang et al., 2015) into bed/bim/fam format.

### 2.2 Computational workflow

Code including the tools sommer and GAPIT, ML algorithms, TSLRF and plotting was run in R Studio server version 2025.5.0.496 with R version 4.4.3 (R Core Team, 2025) on a Ubuntu 22.04.5 LTS server equipped with an Intel(R) Xeon(R) CPU E5–2637 v3 @ 3.50GHz (16 cores, 32 threads). Manhattan & QQ-plots were plotted with ggplot2 version 3.5.2. UpSet plot and Venn diagram were visualized using the R-package ggVennDiagramm version 1.5.2. GCTA, GEMMA and TASSEL were used over the command line. The code (separated in seven R scripts: EN.R [commit tag: 3fcdd80], FeatureSelection [3fcdd80], Gapit.R [3fcdd80], RF.R [2385148], Sommer.R [3fcdd80], TSLRF.R [09f8d51], XBR.R [c20e9f2]) is publicly available on https://github.com/MolinLab/BenchmarkingGWAS. The overall computational workflow is depicted in **Figure 1B** and is given in more detail below:

#### 2.2.1 Traditional GWAS tools

In GAPIT version 3, we applied multiple models including GLM, MLM (Zhang et al., 2010), CMLM (Zhang et al., 2010), SUPER (Wang et al., 2014), MLMM (Segura et al., 2012), FarmCPU (Liu et al., 2016), and BLINK (Huang et al., 2019). In all analyses, two principal components (PCs) were included as covariates and the default settings were used, except for the MAF, which was set to 0.05. For the analysis with sommer version 4.3.6 (Covarrubias-Pazaran, 2016), a standard MLM was used that incorporated a kinship matrix along with two PCs to account for population structure. Similarly, GWAS was performed using GCTA (Yang et al., 2011a), Linux version 1.94.1, employing the mlma option, integrating two PCs as covariates, a genetic relationship matrix, and the MAF was set to 0.05. In TASSEL-5 standalone software, GWAS was carried out using a MLM that also utilized two PCs and a kinship matrix to control for population structure and relatedness. Finally, GEMMA (Zhou and Stephens, 2012) version 0.98.5, Linux was used to perform GWAS under a univariate mixed linear model framework, utilizing a genetic relationship matrix. A common tool for model diagnostics in association mapping is visual assessment of QQ-plots, which can indicate under- and over-powered models with low detection rates or inflated false positive rates (Wang et al., 2022). In addition to visually assessing QQ-plots, we calculated the genomic inflation factor from raw p-values using the following method as described in (Yang et al., 2011b). For well-adjusted models we expect λ = 1, for inflated λ > 1.1 and deflated λ < 0.9.

#### 2.2.2 ML models for GWAS

Machine learning algorithms were implemented using the mlr3 package (Lang et al., 2019) version 0.22.1 using the following extra packages: mlr3learners (Lang et al., 2025) version 0.10.0, mlr3mbo (Schneider et al., 2025) version 0.2.9, mlr3misc (Becker et al., 2025a) version 0.18.0, mlr3tuning (Becker et al., 2025b) version 1.4.0 and mlr3tuningspaces (Bischl et al., 2023) version 0.5.2. Marker selection (feature selection) was utilized using the Elastic Net (EN) algorithm from GLMnet (Friedman et al., 2010) version 4.1–9 included as a standard learner in the mlr3 package in R. Using the best performing model with the following hyperparameters α = 0.6904 and λ = 0.0461 for TKW and α = 0.2298 and λ = 0.1070 for PH. All features with coefficient of 0 were excluded from further analysis which led to 4,018 markers for the TKW dataset and 5,582 markers for PH. EN was also used as a GWAS model. The absolute values of the coefficients of the features were used as importance for this model. Stability selection (Meinshausen and Bühlmann, 2010) was performed as a proxy of feature importance due to the empirical threshold not performing well with the algorithm. RF was implemented using the regression.ranger algorithm from the ranger package (Wright and Ziegler, 2017) version 0.17.0 and decrease of node impurity was selected as feature importance. XGB was performed using xgboost package (Chen et al., 2025) version 1.7.8.1. and feature importance was extracted using gain from the integrated xgb.importance function. Variable importance measure was chosen as a representative for the SVR algorithm. The regression.svm algorithm was used from the R package e1071 (Meyer et al., 2024) version 1.7-16. Feature importance was done by multiplying coefficients with the support vectors. The workflow Two-Stage algorithm based on Least angle regression and Random Forest (TSLRF), which includes population stratification and feature selection, was utilized following Sun et al. (2019). Least angle regression (LARS) (Efron et al., 2004) was applied using Lars package version 1.3 (Hastie and Efron, 2022), RF for TSLRF was applied using the same settings and tuning procedure described for the standalone RF learner. As feature importance metrics, we selected gain for XGB and decrease of node impurity for RF instead of permutation-based importance. Permutation performance performs poorly in the presence of highly correlated predictors, which is a defining characteristic of genomic marker data. In such settings, permuting a single SNP breaks its correlation structure with neighboring loci, causing the model to redirect predictive importance to correlated markers. This leads to unstable, underestimated, and biologically misleading importance estimates. Additionally, permutation importance is most informative when features differ in scale or cardinality, whereas SNP markers all share the same discrete genotype state space (0/1/2).

#### 2.2.3 Hyperparameters and tuning of ML algorithms

For the ML-based GWAS, we adopted a regression framework in which the respective traits (TKW and PH) served as the prediction targets. Since several ML algorithms require numerical or integer matrices as input, we used numerical encoded genomic data generated by GAPIT during GWAS analysis. Hyperparameter ranges were selected using the recommended settings from mlr3tuningspaces for each learner and were subsequently optimized using 5-fold nested cross-validation with Bayesian optimization, also known as model-based optimization. The best hyperparameters were selected by minimizing the cross-validated performance metric, after which the final model was retrained on the full dataset using these optimized hyperparameters (**Supplementary Table 1**). To reduce the impact of algorithmic stochasticity, we retrained the resulting model 100 times, extracted feature-importance scores from each run, and aggregated them to obtain stable estimates.

#### 2.2.4 Significance thresholds

Bonferroni correction (Bonferroni, 1936) was used as a significance threshold and calculated as explained in Wang et al. (2022) for α = 0.05. Additionally, to the Bonferroni correction a false discovery rate (FDR) correction (Benjamini and Hochberg, 1995) with an applied threshold of α = 0.05 was applied. For ML algorithms, an empirical threshold was estimated by a permutation test as described in Wang et al. (2022). Instead of the lowest p-value, we stored the highest variable importance, repeating 1,000 times, and the α = 0.05 percentile as the threshold. Feature importances exceeding this threshold were subsequently treated as significant.

#### 2.2.5 Identifying markers reflecting population structure and relatedness

To assess the influence of population structure on individual markers, we calculated marker informativeness following the approach of Biswas et al. (2009), with the exception that no permutation test was performed. The coefficient of determination (R^2^) values were summed across the first two PCs derived from GAPIT. Higher values indicate stronger influence of population structure on the marker, reflecting its potential association with underlying relatedness patterns.

#### 2.2.6 McNemar’s test for pairwise significance

To assess whether the different models identified significantly different sets of MTAs, we conducted corrected pairwise McNemar’s tests (built-in function in R) using two significance thresholds: α = 0.05 to evaluate overall differences in MTA discovery patterns and α = 0.001 to identify strongly divergent model pairs.

### 2.3 Extracting candidate genes

Linkage disequilibrium (LD) calculated with PLINK 2.0 (Chang et al., 2015) was used to define flanking regions around significant MTAs. Candidate QTLs and genes were identified based on the *Triticum aestivum* reference genome (IWGSC; Ensembl Plants, accessed 18 May 2025; Dyer et al., 2025). Within each defined window, genes were annotated based on gene ontology, functional classification, and prior literature to prioritize and report the most relevant candidate genes.

## 3 Results

All MTAs identified for both traits under different models are listed with their respective candidate genes and functions in **Supplementary Table 2**. An overview of the tools and models used, the number of detected MTAs, computational runtime, and applied significance thresholds is provided in **Table 1**.

**Table 1:** Overview of GWAS models, model form, population structure correction, applied significance thresholds, number of detected marker-trait associations (MTAs), and computational runtime (minutes) for thousand kernel weight (TKW) and plant height (PH).

Tool	GWAS model	Model form	Population structure correction	TKW			PH			Version
				Thresholds	MTAs	Runtime[min]	Thresholds	MTAs	Runtime[min]	
Sommer	MLM	Mixed linear model	2 PCs + Kinship	Bonferroni α=0.05	4	13.0	FDR α= 0.05	2	311.4	4.3.6
GCTA	MLM	Mixed linear model	2 PCs + Kinship	Bonferroni α=0.05	4	1.5	FDR α= 0.05	1	10.0	1.94.1
GEMMA	MLM	Mixed linear model	2 PCs + Kinship	Bonferroni α=0.05	4	3.0	FDR α= 0.05	0	36.0	0.98.5
TASSEL	MLM	Mixed linear model	2 PCs + Kinship	Bonferroni α=0.05	2	1025.0	FDR α= 0.05	4	26220.0	Tassel-5
GAPIT	GLM	Generalized linear model	2 PCs (no Kinship)	Bonferroni α=0.05	1352	8.0	FDR α= 0.05	4482	99.9	Gapit version 3
GAPIT	MLM	Mixed linear model	2 PCs + Kinship	Bonferroni α=0.05	3	26.0	FDR α= 0.05	0	264.2	Gapit version 3
GAPIT	CMLM	Compressed mixed linear model	2 PCs + Kinship	Bonferroni α=0.05	3	1851.0	FDR α= 0.05	2	19551.0	Gapit version 3
GAPIT	MLMM	Multi-locus mixed linear model	2 PCs + Kinship	Bonferroni α=0.05	2	136.8	FDR α= 0.05	2	1435.7	Gapit version 3
GAPIT	SUPER	Mixed linear model with SNP selection	2 PCs + Kinship (selected SNPs)	Bonferroni α=0.05	1101	109.1	FDR α= 0.05	3497	812.6	Gapit version 3
GAPIT	FarmCPU	Iterative fixed and random effects model	Implicit (iterative pseudo-QTNs)	Bonferroni α=0.05	29	1.5	Bonferroni α=0.05	40	4.0	Gapit version 3
GAPIT	BLINK	Fixed-effect multi-locus model	Implicit (LD-based pseudo-QTN selection)	Bonferroni α=0.05	45	2.2	Bonferroni α=0.05	58	4.7	Gapit version 3
TSLRF	EMMA-transformed RF	Random Forest	Kinship	Permutation 1000×, α=0.05 quantile	3	28.9	Permutation 1000×, α=0.05 quantile	0	27.2	0.17.0 (RF), 1.3 (LARS)
RF	Random Forest	Random Forest	None	Permutation 1000×, α=0.05 quantile	44	25.3	Permutation 1000×, α=0.05 quantile	38	343.8	0.17.0
XGB	XGBoost	Gradient Boosting	None	Permutation 1000×, α=0.05 quantile	13	100.6	Permutation 1000×, α=0.05 quantile	10	86.8	1.7.8.1
Elastic Net (EN)	Penalized Regression	Regularized regression	None	Stability Selection 1000x, 95% frequency	6	2.4	Stability Selection 1000x, 95% frequency	21	12.8	4.1.9

The software version used for each tool and model is also reported.

### 3.1 Comparison of traditional GWAS tools

#### 3.1.1 Thousand kernel weight

Most tools evaluated in this study use a Q+K model to identify MTAs, which should result in similar behavior of the tools. However, we observed a different number of MTAs (**Supplementary Figures 1**, **2**): TASSEL found two markers, one on chromosome 2A and three on chromosome 6D, when the Bonferroni threshold was applied. The MLM implemented in GAPIT (**Supplementary Figure 1F**) detected three associations, including one additional marker at chromosome 6D compared to TASSEL. GCTA, GEMMA and sommer highlighted four associations, one at 2D and three at 6D (**Supplementary Figures 1A-D**). Among the MLM approaches, genomic inflation factors varied, ranging from 0.88 for GCTA to 0.99 for TASSEL (**Supplementary Table 3**). This shows that TASSEL is a well-adjusted model, while GCTA, GEMMA and sommer show slightly deflated p-values.

Evaluation of QQ-plots and genomic inflation factors revealed that the models implemented in GAPIT, specifically the GLM and SUPER models, exhibited highly inflated p-values, with genomic inflation factors of 10.7 and 9.7, respectively (**Supplementary Figures 1E**, **2C**; **Supplementary Table 3**). Consequently, these models produced an excessive number of false positives and were excluded from further analysis. In contrast, FarmCPU and BLINK showed a more modest but still significant inflation, with factors of 1.7 and 1.4, respectively (**Supplementary Figures 2D, E**). Although these models will be included in subsequent analyses, their results should be interpreted with caution. The extent of genomic inflation is reflected in the number of MTAs detected: GLM and SUPER identified over 1,000 MTAs, whereas BLINK identified 45 and FarmCPU 29 MTAs (**Supplementary Figure 3A**). CMLM found three and MLMM two marker associations. Two markers, S6D_241296319 on chromosome 6D and S2A_143724068 on chromosome 2A were consistently detected across models (**Supplementary Figure 3A**), underscoring its importance for further investigation. Additionally, FarmCPU and BLINK, both known for finding small-effect loci, shared 14 overlapping MTAs, while FarmCPU found 13 and BLINK 29 unique MTAs (**Supplementary Figure 3A**). These MTAs were detected across almost all chromosomes, namely, 1B, 2A, 2B, 3A, 3B, 3D, 4A, 5A, 5B, 6A, 6B, 6D, 7A, 7B and 7D (**Supplementary Figures 2E, F**).

#### 3.1.2 Plant height

For the plant height (PH) dataset, several MLM-based approaches did not reveal any significant associations when applying a Bonferroni threshold. Only TASSEL, GCTA and CMLM identified significant MTAs at chromosome 7A, while other MLM methods revealed a peak that narrowly missed the threshold (**Supplementary Figure 4**). Using a less stringent FDR threshold, two markers S7A_335064522 and S7A_403940100, were significantly associated across TASSEL, and sommer (**Supplementary Figure 4**). GCTA identified only S7A_335064522, while GAPIT’s MLM and GEMMA reported both markers just below the threshold (**Supplementary Figure 4**). These results highlight how differences in model implementation and computational assumptions can determine whether one, two, or no MTAs are detected. As observed for TKW, GLM, SUPER, FarmCPU, and BLINK exhibited inflated p-values (**Supplementary Table 3**), excluding GLM and SUPER for further analysis. FarmCPU and BLINK showed genomic inflation factors of 2.15 (FarmCPU) and 1.87 (BLINK), identifying a large number of MTAs, 58 for FarmCPU and 40 for BLINK, with an elevated risk of false positives. In contrast, MLM implementations indicated a good model fit, with genomic inflation factors ranging from 0.979 GAPIT to 0.99 GCTA. Among all MLM implementations, TASSEL was the only tool to detect a significant association on chromosome 2B, at an FDR threshold, namely, S2B_770054032 (**Supplementary Figure 4D**). The same marker was also highlighted as significant by FarmCPU and BLINK (**Supplementary Figures 5D, E**).

### 3.2 Comparison of ML-based GWAS approaches

To explore ML for GWAS, we employed the R package mlr3, which provides a robust base set of learners that can be extended with additional packages, e.g. EN, RF, and XGB. SVM was implemented with package e1071, but we encountered a complex prediction error and therefore did not investigate this approach further within this study. Additionally, we evaluated TSLRF, which integrates population stratification using the FASTmrEMMA method (Wen et al., 2018) and feature selection via LARS, followed by a final prediction with a RF model. We acknowledge that our study does not cover all available methods and tools, but the chosen approaches provide a strong foundation for assessing how ML can enhance GWAS in wheat breeding. The selected models provide interpretable feature-importance metrics, which are essential for identifying MTAs. In addition, they represent complementary algorithmic approaches, enabling a diverse yet comparable set of importance metrics that can be directly contrasted with traditional GWAS outputs.

#### 3.2.1 Thousand kernel weight

Interestingly, despite their differing computational complexities and algorithmic approaches, the models yielded very similar RMSE values for the TKW data, suggesting comparable model performance (**Supplementary Table 1**). This finding is somewhat surprising given that each algorithm possesses unique strengths and weaknesses. The real differentiator, therefore, may lie in the features that each model deems important. For EN, six important MTAs were observed on chromosome 1A, 2A, 3D, 5D and 6D (**Supplementary Figure 6D**). The RF model identified 44 highly important markers, with major loci on chromosomes 6A and 6D, and additional MTAs on 2A, 3A, 5A, 6B, 7A, and 7D (**Supplementary Figure 6A**). Similarly, the XGB algorithm highlighted highly important loci on chromosomes 6A and 6D, with additional markers on 1B, 2A, 3A, 5A, 6A, and 7A, resulting in 13 MTAs (**Supplementary Figure 6B**). The TSLRF approach produced results comparable with MLM-based models, identifying three MTAs, one on chromosome 2A and two on chromosome 6D (**Supplementary Figure 6C**).

#### 3.2.2 Plant height

An overview of the outcomes of selected ML models on the PH data is given in **Supplementary Figure 7**. RF found 38 important loci distributed across six different chromosomes (**Supplementary Figure 7A**), however, several markers on chromosomes 5A and 7A were overlapping, suggesting they may represent the same underlying locus. EN found in total 21 important loci on the chromosomes 1B, 2D, 3A, 3D, 4A, 5A, 5B, 6A, 6D, 7A, and 7B (**Supplementary Figure 7D**). XGB highlighted important loci on 2B, 3B, 4A, 4D, 5A, and 7B, but did not identify the major association on chromosome 7A, which was consistently detected by most other models and traditional MLM-based methods (**Supplementary Figure 7B**). Nevertheless, XGB achieved the best model performance in terms of RMSE across ML models for TKW and PH. The TSLRF model, which includes population structure adjustment and feature selection, did not detect any significant associations for PH (**Supplementary Figure 7C**), similar to MLM implementations with Bonferroni correction.

### 3.3 Overlap between traditional and ML methods

#### 3.3.1 Thousand kernel weight

**Figure 2:**
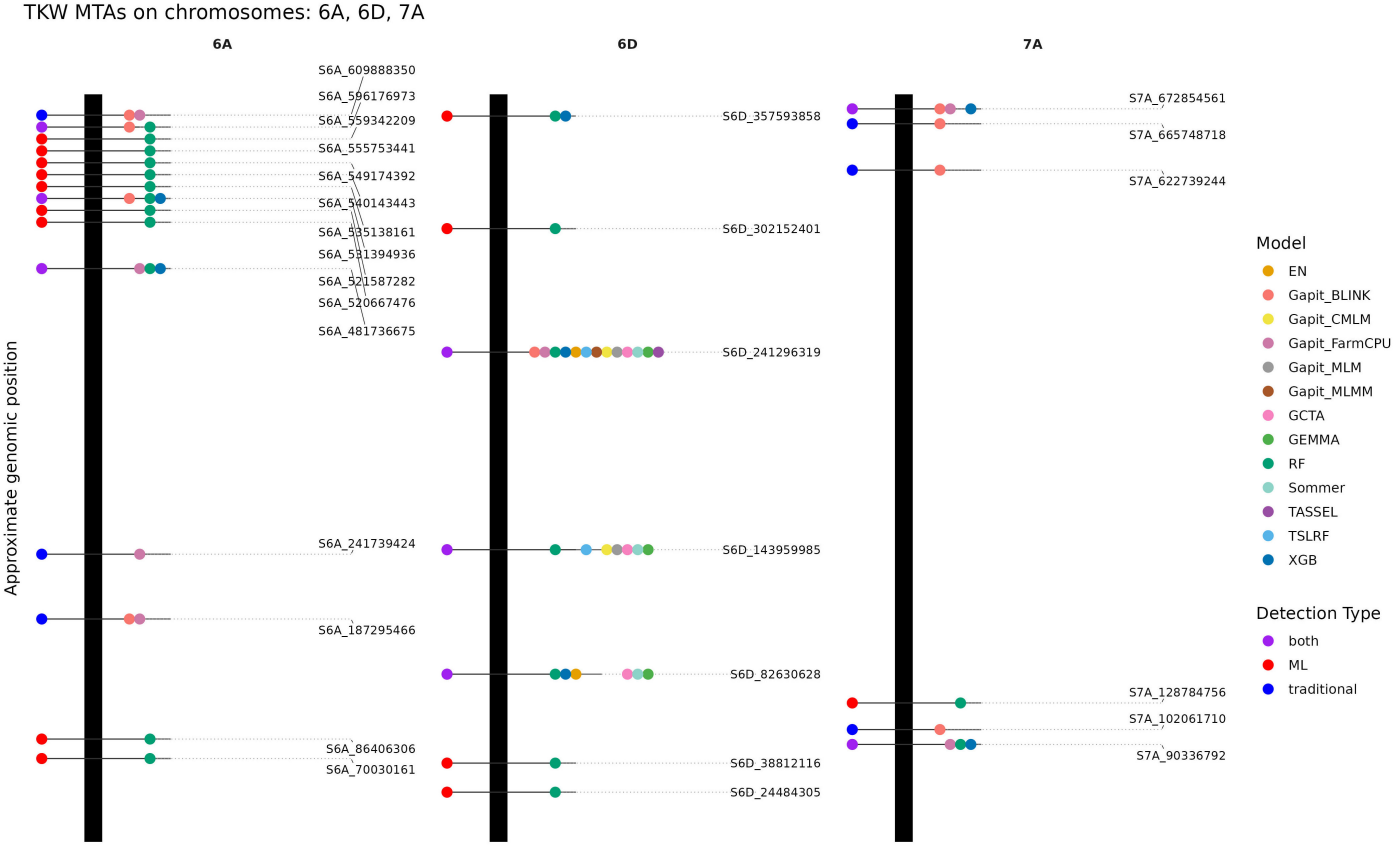
Detected marker-trait associations (MTA) for thousand kernel weight (TKW) identified on the chromosomes 6A, 6D, 7A. The vertical black bars represent the chromosomes and the horizontal lines the approximate position of each associated marker along the chromosome. Each dot on the right side of each chromosome represents MTA detected by one model, with colors indicating the respective method. Dots positioned to the left of each chromosome summarize the detection method by which each MTA was identified.

For TKW, we observed an overlap of significant markers between traditional and ML-based methods (**Supplementary Figures 3A**, **8**-**10**). For example, S6D_241296319 was found using all models, or S6D_143959985 that was also significantly associated using the models CMLM, MLM (using GAPIT, GCTA, GEMMA, sommer), TSLRF and RF (**Figure 2**). While S2A_143724068, which was found to be associated with TKW using multiple traditional methods, was only significantly associated using the EN and TSLRF model (**Supplementary Figure 8**). However, RF detected S2A_143241161 in proximity, suggesting detection of the same biological signal. Another key marker, S6D_82630628, was consistently associated using the MLM from GCTA, GEMMA and sommer as well as with EN, RF, and XGB (**Figure 2**). Using the genomic inflation value, we were able to estimate the model performance in terms of inflation of p-values. BLINK and FarmCPU showed inflated p-values, which suggests a more careful interpretation of associated markers using these models. Some of these markers were, however, also highly important using ML models: S1B_642678709 (FarmCPU, BLINK, XGB), S3A_642133365 (FarmCPU, BLINK RF, XGB), S3D_181564815 (FarmCPU, BLINK, EN), S5A_561661320 (BLINK, RF, XGB), S6A_481736675 (FarmCPU, RF, XGB), S6B_157666111 (FarmCPU, BLINK, RF), S7A_672854561 (FarmCPU, BLINK, XGB), and S7A_90336792 (FarmCPU, RF, XGB) (**Supplementary Figures 8**-**10**). Therefore, these markers, which would not have been included using the best-fit MLM, should still be investigated due to their relevance across ML algorithms. In total, 96 unique MTAs associated with TKW were detected across all evaluated models, excluding GLM and SUPER due to high inflation rates (**Supplementary Table 2**).

#### 3.3.2 Plant height

**Figure 3:**
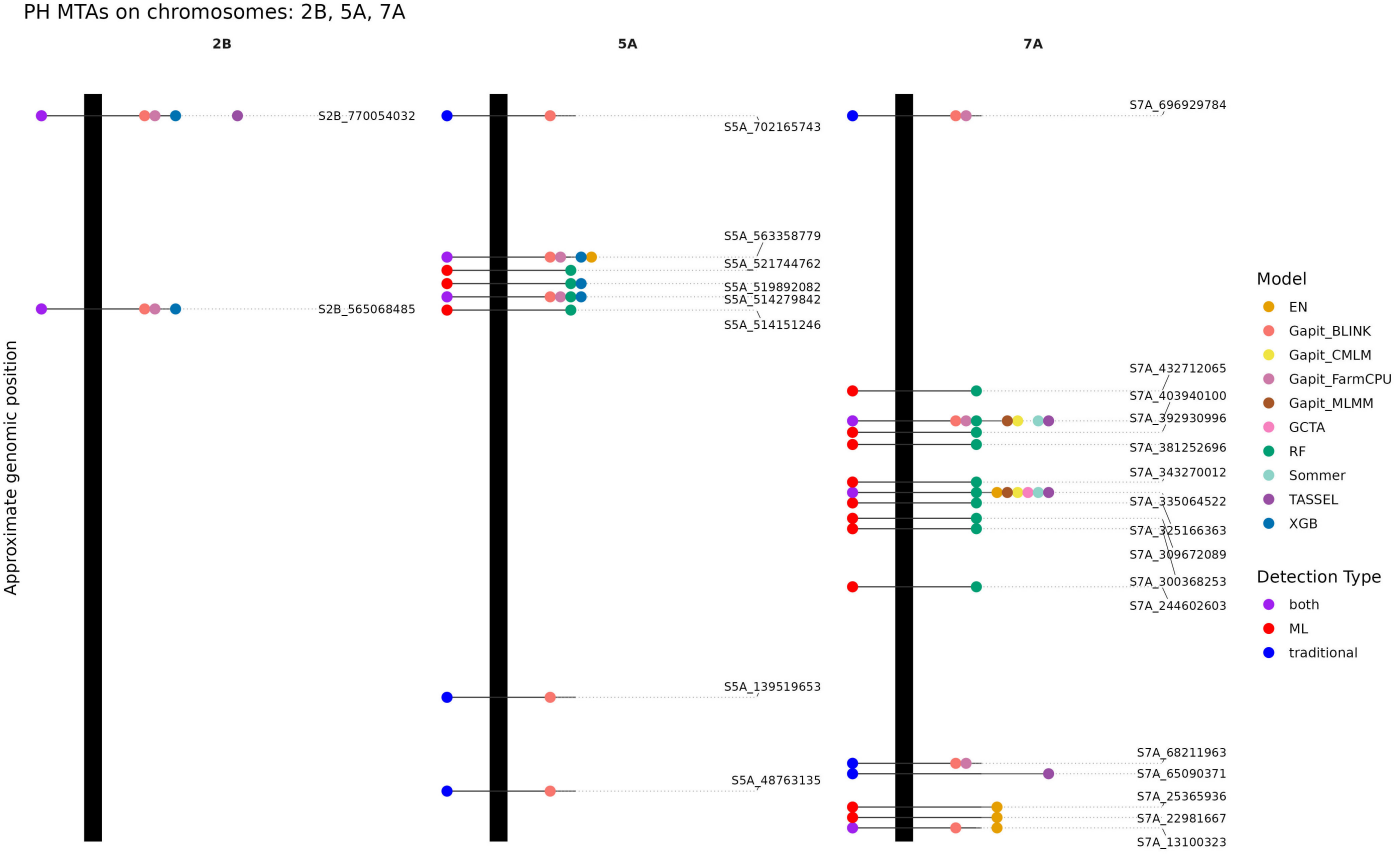
Detected marker-trait associations (MTA) for plant height (PH) identified on the chromosomes 2B, 5A, 7A. The vertical black bars represent the chromosomes and the horizontal lines the approximate position of each associated marker along the chromosome. Each dot on the right side of each chromosome represents MTA detected by one model, with colors indicating the respective method. Dots positioned to the left of each chromosome summarize the detection method by which each MTA was identified.

When comparing the detected MTAs for PH, no single marker was identified by all models (**Supplementary Figures 3B**, **11**-**13**). However, two markers were notable due to appearance across approaches: S7A_403940100 and S7A_335064522, each detected by six different models (**Figure 3**). Interestingly, the latter marker was not found in FarmCPU and BLINK. Other markers also showed cross-method detection. For example, 3B_757480752 and S5A_514279842 were consistently found using FarmCPU, BLINK, RF, and XGB (**Figure 3**; **Supplementary Figure 12**). Given the inflation of the p-values, their repeated detection by both traditional and ML models verifies the importance of these loci. Several further loci demonstrated high importance across three models: S2B_565068485 was found by FarmCPU, BLINK, and XGB, while S1B_568961025 was detected by FarmCPU, BLINK, and RF (**Figure 3**; **Supplementary Figure 11**). Similarly, S3D_282956863 and S3D_529775477 were jointly identified by FarmCPU, BLINK, and EN, suggesting consistent signals across parametric and regularized regression models (**Supplementary Figure 11**). S4A_679187682 was detected by the decision tree models RF and XGB, while also being significantly associated using BLINK. Likewise, S5A_563358779 was identified using four different methods, namely, FarmCPU, BLINK, EN and XGB. S2B_770054032 was found by TASSEL’s MLM implementation as well as by FarmCPU, BLINK, and XGB. In total, 119 unique MTAs were identified across all models except GLM and SUPER, indicating substantial discovery power when combining traditional and ML-based methods (**Supplementary Table 2**).

### 3.4 Pairwise comparison

Using McNemar’s test (**Supplementary Figures 14**, **15**), we assessed whether the different models identified significantly different sets of MTAs. For TKW, the results revealed a clear methodological separation among the approaches. The MLM-based approaches, together with TSLRF and EN, showed no significant pairwise differences, indicating that these methods detected largely overlapping MTAs. In contrast, FarmCPU, BLINK, RF, and XGB produced MTA sets that differed significantly from the MLM cluster. Within this non-MLM group, BLINK and RF as well as RF and FarmCPU did not differ significantly, whereas most other comparisons were significant. Notably, XGB and EN did not differ significantly, suggesting partially overlapping signals despite their methodological differences. Importantly, XGB differed from most MLM approaches only at α = 0.05, whereas FarmCPU, BLINK, and RF showed much stronger divergence at α = 0.001.

For PH, a similar pattern emerged. The MLM methods that detected MTAs showed no significant pairwise differences, while all other models yielded MTA sets significantly different from the MLM cluster, with the exception of TASSEL, which did not differ from XGB. Again, XGB and EN showed no significant difference, although EN differed significantly from all MLM approaches for PH, in contrast to its alignment with MLM methods for TKW. FarmCPU and BLINK did not show similarity to any other model, with the only non-significant comparison observed between RF and FarmCPU. Similar to the TKW results, XGB differed from MLM approaches only at α = 0.05 for PH, except for the comparison with TASSEL, which was not significant. Influence of population structure on MTAs.

#### 3.4.1 Thousand kernel weight

Due to the high inflation rates observed in FarmCPU and BLINK (**Supplementary Figures 2D, E**), we investigated marker informativeness along the PCs to estimate if the described MTAs (see **Figure 3**) might be confounded by population structure. However, associations with population structure alone cannot distinguish between effects driven by demographic history and those potentially shaped by selection. To further explore possible confounding, we overlaid MTAs detected by both the traditional and ML-based models and evaluated whether significant associations were disproportionally enriched among SNPs with high structure-related variance.

Across the genome, MTAs were distributed over a wide range of informativeness values, indicating that most associations are not primarily driven by markers that strongly covary with population structure. Furthermore, no systematic differences were observed between traditional and ML-based approaches in how their detected MTAs aligned with the informativeness spectrum, with the exception of chromosome 6A, where a cluster of MTAs detected exclusively by RF ranked relatively high, whereas MTAs detected by both methods showed lower informativeness values.

**Figure 4:**
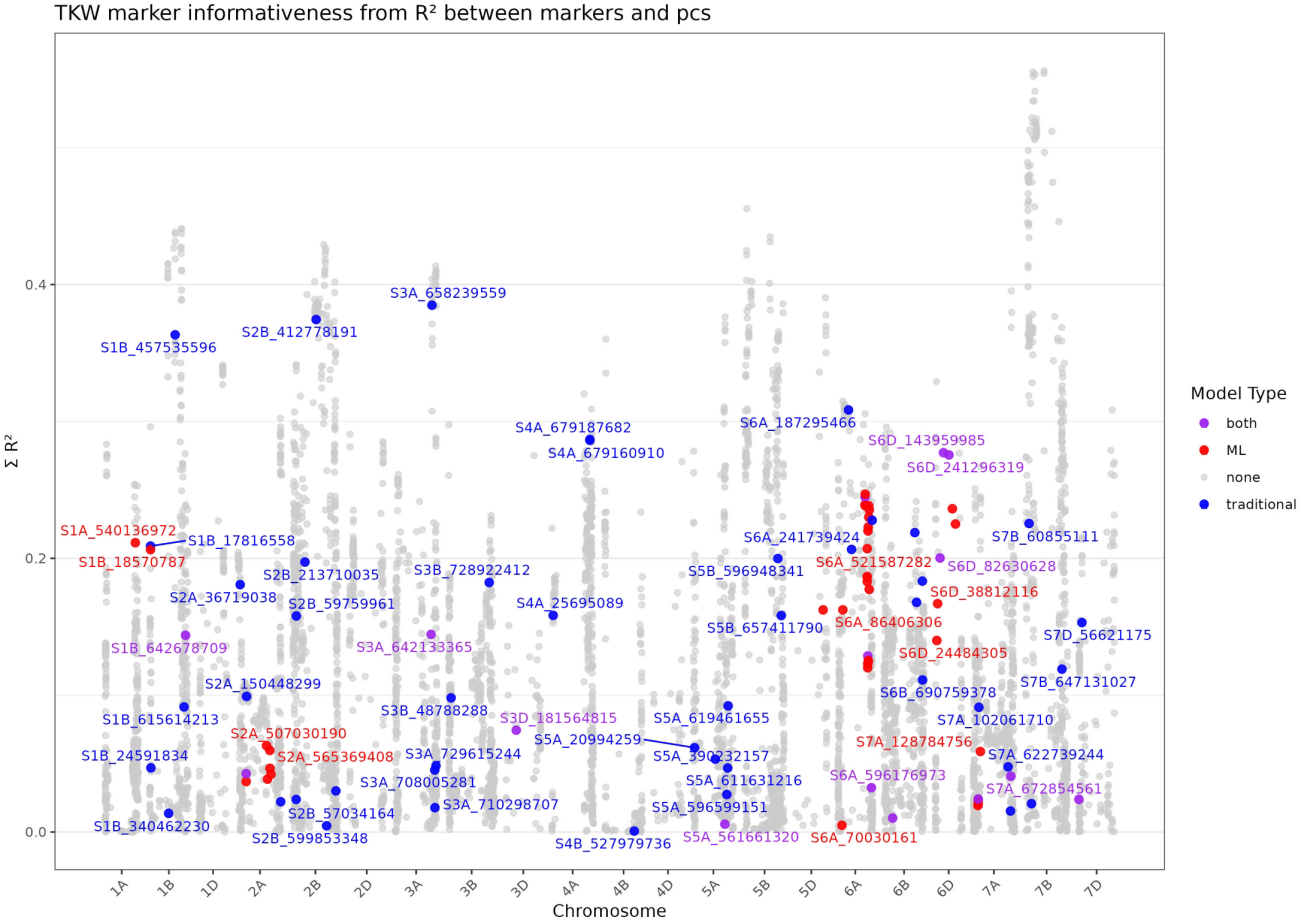
Marker informativeness based on the coefficient of determination (R²) between all markers and the first two principal components for thousand kernel weight (TKW). The y-axis shows the summed R² values across the first and second principal components, The x-axis represents the genomic position of markers across chromosomes. Marker–trait associations (MTAs) are highlighted according to the model that detected them: blue for traditional GWAS models, red for ML–based models, and purple for markers detected by both approaches.

Additionally, we identified several MTAs with high informativeness values > 30%, suggesting potential confounding (**Figure 4**). These include S1B_457535596 and S2B_412778191, detected exclusively by BLINK, and S3A_658239559 and S6A_187295466, detected by both FarmCPU and BLINK. Other markers such as S4A_679160910 and S4A_679187682, detected by FarmCPU and BLINK, respectively, ranked relatively high explaining ~29%, which is around 10% less compared to the top-ranked MTAs on 1A, 2B and 3A. Additionally, S6D_143959985 and S6D_241296319, detected by multiple models including MLMs, explained 28%.

#### 3.4.2 Plant height

Overall, MTAs for PH were distributed across a wide range of cumulative variance, indicating, similar to TKW, that most associations are not closely aligned with markers that strongly correlate with population structure. Instead, the majority of MTAs, regardless of the method used, exhibited relatively low informativeness. Interestingly MTAs detected by ML-based methods ranked comparatively lower than those identified by traditional methods although some MTAs detected by both methods ranked higher.

**Figure 5:**
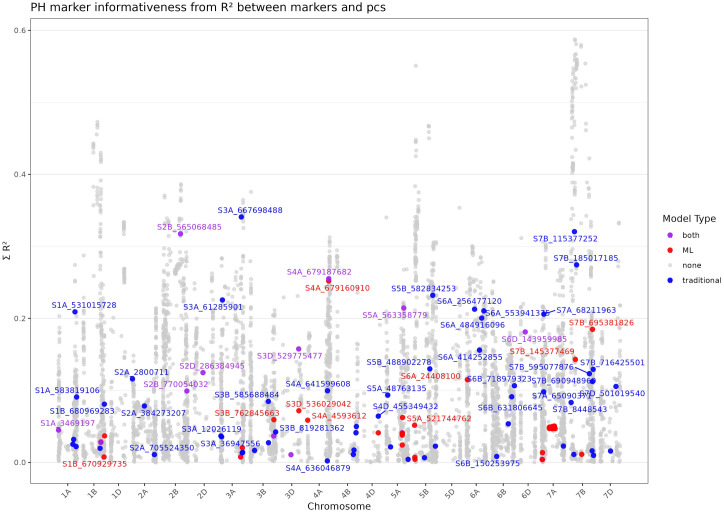
Marker informativeness based on the coefficient of determination (R²) between all markers and the first two principal components for plant height (PH). The y-axis shows the summed R² values across the first and second principal components. The x-axis represents the genomic position of markers across chromosomes. Marker–trait associations (MTAs) are highlighted according to the model that detected them: blue for traditional GWAS models, red for ML–based models, and purple for markers detected by both approaches.

The highest-ranking MTAs for plant height, were S2B_565068485 identified by FarmCPU, BLINK and XGB, S3A_667698488 (FarmCPU and BLINK), S7B_115377252 (BLINK), each explaining more than 30% of variation individually (**Figure 5**). Other noteworthy markers include S4A_679160910 (RF) and S4A_679187682 identified by BLINK, XGB and RF as well as S7B_185017185 detected by BLINK. It is important to note that no thresholds or p-values were calculated to define markers as significantly associated with population structure. Therefore, these results should be interpreted as indicative rather than definitive.

### 3.5 Computational efficiency

In terms of computational efficiency on the Intel(R) Xeon(R) CPU E5–2637 v3 @ 3.50GHz (16 cores, 32 threads), the tools demonstrated considerable variation. It is important to note that the server was used in parallel by multiple users during these experiments, which may have introduced variability in the reported computation times due to resource contention. Under these conditions, TASSEL exhibited the longest total computation times, ~1,025 minutes (min) for TKW and 26,220 min (~ 18 days) for the PH. GAPIT showed large variation across models: for TKW, runtimes ranged from 1.5 (FarmCPU) to 1,851 min (CMLM), and for PH, from 4 min (FarmCPU) to 19,551 min (CMLM). Notably, FarmCPU and BLINK consistently ran significantly faster than other GAPIT models. Sommer completed in 13 min (TKW) and 311 min (PH). Command-line tools such as GCTA required ~2 min (TKW) and 10 min (PH), while GEMMA took 3 min (TKW) and 36 min (PH). For the ML models implemented in this study, computational times included both hyperparameter tuning and model training, and were strongly influenced by the chosen hyperparameters. In general, the models ranked from fastest to slowest as follows: EN (TKW: ~ 2 min; PH: ~ 12 min) < RF < XGB (TKW: 100 min; PH: 344 min). EN was the fastest, which may be attributed to its efficient regularized regression framework. RF and XGB, on the other hand, incurred higher computational costs, partly due to hyperparameters that were computationally intensive. For instance, we used 1,000 rounds of boosting for XGB, and reducing this number would decrease computational time but would result in less performance.

## 4 Discussion

The overall objectives of this study were to compare traditional and ML-based GWAS tools and methods amongst and across each other and highlight the potential of integrating ML to enhance the characterization of marker-trait associations by focusing on wheat as an important staple crop. Only a few studies attempted similar comparisons while some focusing only on traditional GWAS applications (Galesloot et al., 2014; Saber and Jesse Shapiro, 2020; Yang et al., 2024), others compared statistical GWAS models (Wen et al., 2018; Kaler et al., 2020; Malik et al., 2021; Merrick et al., 2022; Sandhu et al., 2024), and others incorporated ML-based methods (Gill et al., 2022; Yoosefzadeh-Najafabadi et al., 2022). However, to the best of our knowledge, there is only one study to date that applied several different ML algorithms in wheat, extracted feature importance metrics, calculated an empirical cutoff, and directly compared their findings with traditional approaches (Tekeu et al., 2023). But their analysis was conducted on a relatively small dataset (170 samples) and did not include well-established learners such as XGB and EN. Thus, in our study, we aimed for a higher sample number by using the well-known and publicly available CIMMYT dataset (Juliana et al., 2019) by focusing on the traits plant height (PH, 7,886 samples) and thousand kernel weight (TKW, 3,478 samples) in wheat, assessing both statistical results with detected MTAs and the practical usability of different ML-based models. Since MAS continues to play a central role in breeding programs aimed at improving wheat, research into ML-based GWAS is particularly relevant for this crop.

### 4.1 Evaluation of traditional GWAS tools

Even though most of the traditional tools primarily rely on a MLM, subtle differences in implementation and internal computations resulted in variations in significance levels. In most cases, these differences were minor and led to a few MTAs falling just above or below the significance threshold depending on the tool used. However, the biggest distinction across tools was computational performance and model flexibility. TASSEL provided an easy-to-use platform with several in depth filtering options, making it a solid choice for pre-processing, SNP filtering, and imputation, and has been used already several times in wheat GWAS (e.g. Sukumaran et al., 2015; Liu et al., 2017; Genc et al., 2019).

However, in our study, TASSEL exhibited notably long computation times, including a runtime of up to 2.5 weeks for the PH dataset. While this may partly reflect less efficient computation and parallelization constraints, it is important to consider that the server was shared among multiple users, which could have influenced the observed runtimes. TASSEL does offer additional modelling options, such as GLM and Fast Association, which can significantly increase computation time but may not always adequately account for kinship and population structure. Sommer, implemented in R, provides users with the flexibility to define complex MLM structures and random effects tailored to experimental designs. While its runtime was moderate in our tests, its customizability and compatibility for HapMap-formatted genotype data make it a valuable tool. GCTA and GEMMA, both command-line tools optimized for PLINK input, consistently demonstrated faster performance compared to TASSEL and R-based tools. However, converting HapMap files into the required input format may present a challenge for some users. Among the tools evaluated, GAPIT offered the widest range of model options, allowing users to select the best model based on genomic control or benchmark results. Computational times varied significantly across GAPIT models, with FarmCPU and BLINK running considerably faster than others. While CMLM was introduced to reduce computational time (Zhang et al., 2010), the process of calculating the optimal compression level, such as the 158 grouping variations for PH, substantially increased runtime compared to other GAPIT models. This observation might be data size-dependent and could be more pronounced with datasets containing several thousand individuals.

GAPIT’s variety also highlights how different model assumptions influence results. For instance, the basic GLM tests one marker at a time with only population structure covariates without kinship matrix, to account for relationships among individuals. As a result, GLM often produces many spurious associations when individuals are related, or population structure is complex (Yu et al., 2006). This behavior is reflected in our study and other experiments and simulations (Kaler et al., 2020; Sandhu et al., 2024). In SUPER, kinship is built from a subset of informative markers, and markers in LD with each test SNP are excluded to avoid confounding (Liu et al., 2016). However, using only a few pseudo-QTNs often fails to capture the full relatedness structure, reintroducing confounding. Thus, SUPER can still suffer high inflation if the pseudo-QTNs do not fully account for cryptic kinship, especially in structured wheat populations. The high amount of spurious associations we observed is in line with simulation studies (Wen et al., 2018; Kaler et al., 2020; Sandhu et al., 2024). MLM approaches i.e. MLM, CMLM, MLMM performed well in controlling for population stratification and relatedness which is reflected in the genomic inflation values. However, this strict control comes at the cost of power to detect true associations (Kaler et al., 2020; Huang et al., 2019). By modelling the polygenic background, MLM-based tests effectively attribute variance to kinship and leave little for each SNP. As a result, only very strong MTAs will pass strict significance thresholds (Liu et al., 2016). Simulations under high heritability showed that MLM/CMLM detected five out of 20 true QTLs, while MLMM was able to find 11 out of 20 (Sandhu et al., 2024). This result is mirrored in the results for the trait TKW in this study. No association was detected for PH using GAPIT’s MLM approach, which could be explained by overfitting of kinship. Although when using different clustering in CMLM, the model detected one MTA, showing a slight increase in power. FarmCPU and BLINK showed moderately inflated λ, which might reflect spurious signals. Empirically, FarmCPU often finds more associations than MLM but allows for more false positives, while BLINK efficiently detects associations often with less false positives, outperforming FarmCPU (Huang et al., 2019). When assessing potential confounding due to population structure, we observed that certain MTAs uniquely detected by FarmCPU and BLINK indeed showed higher informativeness values, indicating a greater likelihood of being influenced by population structure. This may reflect differences in the modeling approaches of these methods and their interaction with the specific characteristics of the analyzed dataset. Nevertheless, not all MTAs detected by BLINK and FarmCPU appear to be related due to population structure. The higher number of significant markers observed from these methods likely arises due to application of multi-locus tests in FarmCPU and BLINK, enabling detection of loci that single-marker tests might miss (Merrick et al., 2022). However, the potential of false positives, as indicated by the model inflation underscores the value of incorporating ML approaches as a benchmark to further validate and refine these findings.

### 4.2 Evaluation of ML-based models for GWAS

The genetic architecture of the traits TKW and PH in wheat is clearly more complex than can be captured by purely linear, single marker models. In order to account for the well‐known limitations of traditional GWAS, namely their inability to capture non-linear and epistatic interactions, we employed ML-mediated GWAS to complement conventional methods. Yoosefzadeh-Najafabadi et al. (2022) demonstrated that ML-based GWAS can enhance the detection of MTAs in soybean, providing a broader and more comprehensive genetic insight than conventional methods alone.

Unlike traditional models, ML approaches do not estimate direct allele substitution effects. Instead, we focused on their capacity to identify MTAs, QTLs, and candidate genes. To further assess their predictive performance, we evaluated overall model fit using RMSE, where XGB outperformed other models for both traits, PH and TKW. TSLRF was developed with the same reasoning as MLMs, that control for population structure and polygenic background, which is crucial to reduce false positives, i.e. importance rankings of SNPs (Price et al., 2006; Sun et al., 2019). Previous work by Stephan et al. (2015) showed that RF and Lasso correcting for population structure outperformed their unadjusted counterparts. However, this stringent control might come at the expense of power, which is reflected in high false negative rates of MLM (Liu et al., 2016). This trade-off was evident in our results: for TKW, TSLRF only detected markers on chromosomes 2A and 6D, consistent with MLM findings. Notably, eight of the ten most important markers overlapped with MTAs from other models, suggesting that a less stringent cutoff might reveal additional meaningful associations, similar to traditional approaches. For PH no marker was significantly associated which is consistent with some MLM approaches used in this study and further supports the notion that stringent control may decrease detection power in traits with more complex architectures.

In contrast, RF without a control for population structure and relatedness, identified a larger number of MTAs. This notable difference to TSLRF indicates that some of the MTAs detected may partly reflect confounding due to population structure. However, when investigating marker informativeness along the PCs this does not seem to be the dominant cause. Another factor could be that, unlike EN and XGB, RF lacks feature regularization resulting in correlated markers receiving similar importance scores. This is more likely, especially because most MTAs identified in RF cluster at Chromosome 6A for TKW and 5A and 7A in PH. Investigation of these clusters revealed that most of the markers are in strong LD, thereby inflating the number of associations. This discrepancy to EN and XGB suggests that regularization might be very important for ML-based GWAS approaches. Despite these limitations, the unadjusted RF model was able to detect MTAs with showed strong biological relevance, with a pipeline that better accounts for correlated features, RF might be a powerful algorithm for GWAS. Previous studies showed the high potential of RF in detecting MTAs: both, RF and gradient boosting machines identified regions with multiple candidate genes and some previously not reported in cattle (Alves et al., 2022; Li et al., 2018). In soy, RF detected more regions compared to traditional models (MLM and FarmCPU) for yield component traits, with no overlap between the approaches (Yoosefzadeh-Najafabadi et al., 2022). In contrast, our study revealed partial overlap between RF and traditional approaches, suggesting that while RF uncovers additional loci, it can still capture some of the core signals detected by traditional GWAS methods.

XGB, designed for high-dimensional and complex data, is well-suited for GWAS applications. Gill et al. (2022) showed that XGB not only outperformed deep learning architectures on most traits but also detected loci associated with flower and seed coat color that are supported by previous research. For complex traits with non-additive gene action gradient boosting performed better than traditional RF and deep learning architectures (Abdollahi-Arpanahi et al., 2020). In our study, compared to mixed‐model single‐marker tests, XGB identified more MTAs, but found fewer than multi‐locus methods like FarmCPU and BLINK. Due to efficient regularization XGB was able to better able to rank markers compared to RF. For TKW, 13 MTAs were detected, seven of which overlapped with those detected by RF. Notably, three MTAs were exclusively identified by these ML algorithms, two of which were linked to biologically plausible gene candidates, highlighting the potential of ML to uncover possible non‐linear or epistatic interactions overlooked in single‐marker mixed models.

The regularized regression model GLMnet is commonly used for feature ranking (Dagasso et al., 2021) or feature selection (Cho et al., 2010; Waldmann et al., 2013). John et al. (2022) compared several ML algorithms on prediction and feature selection performance and showed that EN performed as one of the best selection algorithms with good prediction performance outperforming other ML algorithms. However, EN reliance on regularization can lead to unstable features and may fail to select all informative features. To address this, Meinshausen and Bühlmann (2010) proposed stability selection, a procedure that aggregates selection results across subsamples to improve consistency and control false discoveries. Alexander and Lange (2011) investigated this process on the Wellcome Trust Case-Control Consortium human dataset, however, in this case Lasso was used instead of EN, finding that stability selection effectively controls the family-wise error rate but suffers from a loss of power. Nevertheless, in our study, we implemented stability selection alongside EN because the ridge component of EN preserves groups of correlated predictors, resulting in a less sparse model, while stability selection further improves the robustness and interpretability of our marker rankings. Although EN does not model non‐linear or interaction terms directly, it uncovered several distinct MTAs linked to genes associated with the traits. For PH, a notable example was S7A_22981667, which neither tree‐based methods nor MLMs detected. These unique SNPs likely represent additive effects that were too subtle to surpass genome-wide thresholds in single-marker MLMs yet survived the regularization and stability‐selection process.

Overall, ML-based approaches recovered core MTAs identified by traditional GWAS while differing in their sensitivity to additional loci, with overlap patterns strongly dependent on the specific algorithm and trait. Interpretations involving MLM-based comparisons should nevertheless be made cautiously, as the small number of detected MTAs resulted in sparse contingency tables and limited power, despite the use of a continuity-corrected McNemar’s test.

### 4.3 Functional characterization of shared MTAs identified by traditional and ML-based GWAS

To assess the added value of using ML approaches for GWAS and candidate gene discovery, we examined the MTAs previously identified by ML approaches for their biological function relevant for the analyzed traits. After removing MTAs in strong LD, we found 21 markers with relevant genes, 11 for TKW and ten for PH. First, we will discuss those MTAs that were found in traditional methods and further validated using ML-based GWAS approaches.

The TKW-associated marker S2A_143724068 was identified by all models except RF and XGB and points to the candidate gene *TraesCS2A02G183900*. This gene was previously reported by Liu et al. (2022) as an auxin-responsive differentially expressed gene involved in phytohormone metabolism and signaling in pairwise near-isogenic lines. Auxin plays a critical role in lateral root initiation and development. Root spatial configuration in the soil is recognized as a promising strategy to enhance crop yield (Lynch, 2022). Following this reasoning it might be a promising candidate for TKW. Marker S3D_181564815 (FarmCPU, BLINK, EN) co-localizes with three candidate genes potentially influencing TKW. The first one, *TraesCS3D02G191300* encodes an expansin, a cell-wall–loosening protein promoting cell expansion in growing tissues such as the developing endosperm. At the molecular level, expansin expression is a key determinant of grain cell enlargement and thus final kernel size. Although 241 expansins have been annotated in wheat, only a subset has been functionally characterized, with some linked to grain traits (Mira et al., 2023). *TraesCS3D02G189900*, a Class III peroxidase, modulates cell-wall stiffening and has been associated with spikelet traits inversely related to TKW. Overexpression of the related gene *TaPRX-2A* reduced grain number per spike and spike length, indicating potential pleiotropic effects (Zang et al., 2025). Additionally, *TraesCS3D02G189500*, a putative nitrate transporter (NPF family), is implicated in nitrogen use efficiency which is a key determinant of grain filling and starch accumulation (Kumar et al., 2022). The gene *TraesCS5A02G359900*, annotated as a serine/threonine protein phosphatase, was the most likely candidate for marker S5A_561661320 (Blink, RF and XGB). Hu et al. (2012) identified a serine/threonine protein phosphatase gene as a candidate after fine mapping of a grain length, grain width and TKW QTL in rice. A promising candidate for marker S6A_481736675 (FarmCPU, RF, XGB) is *TraesCS6A02G259000*, a MADS-box transcription factor serving as a candidate gene for grain size and weight in *Triticum turgidum* (Mangini et al., 2025). Marker S6A_484065257 (FarmCPU, RF, XGB) lies near *TraesCS6A02G260600* and *TraesCS6A02G260500* that share a seed−storage helical domain and are annotated for lipid transport (GO:0006869). These non-specific lipid transfer proteins have been linked to cuticle formation and seed coat integrity and knock-out plants in rice showed decreased TKW and grain size (Li et al., 2023b). Located near marker S6A_531394936 (BLINK, RF, XGB) is *GS1* (*TraesCS6A02G298100*), a central enzyme in the glutamine synthetase (GS)/glutamate synthase (GOGAT) cycle which is involved in N assimilation and represents a crucial metabolic step in regulating nitrogen use efficiency and grain yield (Fortunato et al., 2023). Additional studies on the GS1 gene family in several crops have shown that they play different roles in determining seed size and number, grain filling and development (Tabuchi et al., 2005; Bernard et al., 2008; Fortunato et al., 2023). In the LD interval also lie *TraesCS6A02G296500* (Manjunath et al., 2023) and *TraesCS6A02G296400* (Devate et al., 2023), which have been reported as QTLs for TKW. Marker S6D_82630628 (GCTA, GEMMA, sommer, RF, XGB, and EN) falls directly on *TraesCS6D02G116200* (*TaPRX-2A*), encoding a Class III Peroxidase gene previously shown to regulate grain number per spike in common wheat (Zang et al., 2025). Because grain number per spike and TKW are often negatively correlated, variation in *TaPRX-2A* may indirectly influence TKW. Similarly, *TraesCS7A02G136600*, a COBRA-like protein showing a range of functions and participate in various developmental processes in cereals. In rice, for example, it has been associated with QTLs for grain number per spike (Mizuno et al., 2021). While this gene also showed no direct correlation with TKW, the study highlighted the complex genetic architecture in spike traits. S7A_90336792 linked with *TraesCS7A02G136600* was significantly associated with FarmCPU, RF and XGB.

Regarding the trait PH, the marker S5A_563358779 (FarmCPU, BLINK, XGB, EN), which corresponds to a cluster of Cytochrome P450 (CYP450) genes (*TraesCS5A02G362400*, *TraesCS5A02G361800*, *TraesCS5A02G361700*, *TraesCS5A02G363000*, *TraesCS5A02G362900*, *TraesCS5A02G362600*, *TraesCS5A02G362700*), has been detected in our study. Some members of the CYP450 family play a key role in brassinosteroid biosynthesis, which influences plant height through the regulation of cell elongation. The biosynthesis and catabolism of brassinosteroids rely on the enzymatic activity of several CYP450 subfamilies (Li and Wei, 2020). Manipulating the expression of BR-related genes can result in pleiotropic effects, some of which may be undesirable in crop breeding. For example, changes in BR pathways often lead to altered plant architecture, including reduced or excessive plant height (Yamamuro et al., 2000). Furthermore, the markers S1B_568961025 (FarmCPU, BLINK, RF) and S7A_403940100 (BLINK, FarmCPU, MLMM, sommer, TASSEL_MLM, RF) lie close to a Growth-Regulating Factor (GRF) and zinc finger GRF, respectively. GRFs are plant-specific transcription factors known to play critical roles in plant growth, development, and responses to environmental stress (Wu et al., 2014; Van Der Knaap et al., 2000; Bazhenov et al., 2021), demonstrating the essential role of GRFs as candidate genes for PH.

The high amount of overlapping markers shows ML-driven GWAS can effectively validate and prioritize potential MTAs that are detected in traditional methods like FarmCPU and BLINK, which show a higher power to detect associations at a cost of more false positives. However, in addition to MTAs that co‐localize with genes of known function, we also identified several associations without any obvious candidate annotation. Two of these unannotated loci stood out for their consistency across methods: S6D_241296319 associated with TKW was identified by all approaches and S7A_335064522, associated with PH detected by six methods. These findings reflect the fact that GWAS is only as informative as the available functional annotation, and without high‐confidence gene models or known pathways, the biological interpretation remains uncertain.

Notably, the marker S2B_565068485, which was detected by FarmCPU, BLINK, and XGB, showed no obvious candidate genes in its vicinity but instead exhibited a signal pattern more closely aligned with population structure, suggesting potential confounding. A similar pattern was observed for S4A_679187682, identified by BLINK and both tree-based learners. These findings indicate that ML-based GWAS can be susceptible to residual confounding when stratification is not accounted for. However, some markers, for instance, S6D_143959985 or S6D_241296319 identified by most approaches applied in this study including the conservative MLM showed similar patterns. A likely conclusion might be that at least some of these signals may reflect genuine selection-driven associations rather than structural or noise-related artifacts.

### 4.4 Unique MTAs revealed by ML-based approaches

While the overlap between ML-based and traditional GWAS highlights the robustness of the detected MTAs, we now focus on the ability of ML algorithms to detect additional, previously overlooked associations to extend and enrich conventional discovery pipelines, building on prior studies that have already demonstrated this potential (Mieth et al., 2021; Alves et al., 2022; Gill et al., 2022; Ghose et al., 2024).

Tree-based ML algorithms uncovered three novel MTAs that were not detected by traditional GWAS. For TKW, a candidate behind S2A_507030190 (RF, XGB) is *TraesCS2A02G294600*, blue‐light inhibitor of cryptochromes *1 (BIC1)*, functioning as a transcriptional coactivator that promotes brassinosteroid signaling and plant growth (Yang et al. 2021b). Since brassinosteroids might directly influence grain size through cell‐expansion pathways, this MTA represents a physiologically plausible contributor to TKW variation. For PH, S5A_519892082, found by RF and XGB, and S4D_455321401, solely found by XGB, showed several promising genes in their proximity: the first marker is near *TraesCS5A02G307300*, a PHD-type domain-containing protein, playing an important role in plant growth, development and response to abiotic stress (Pang et al., 2022) and two CYP450 genes (*TraesCS5A02G307700*, *TraesCS5A02G307600*) linked to PH. The second marker is associated with TraesCS4D02G283000, a MYB transcription factor, playing important roles in plant development, including internode elongation and stem architecture. Although relatively few studies have directly linked MYB transcription factors to stem development, available evidence suggests their involvement in regulating stem height and strength. For instance, overexpression of *TaMYB18s* led to a pronounced leaf rolling phenotype, a phenotype also related to drought stress (Kadioglu and Terzi, 2007), and a significant reduction in plant height in wheat (Zhang et al., 2016). Similarly, *GmGAMYB* has been shown to promote stem elongation via the gibberellin signaling pathway in soybean (Yang et al., 2021a), while *OsMPH1* acts as a positive regulator of stem height in rice (Zhang et al., 2017). These findings support the potential involvement of MYB-related genes in regulating plant height across different species. On the other hand, by applying EN, most MTAs for TKW overlapped with markers that were found with traditional methods, while also finding two unique markers. One of them, S1A_540136972, is located near the gene *TraesCS1A02G357800*, which is involved in the regulation of flower development (GO:0009909). This gene belongs to the AP2/ERF transcription factor family, previously shown to increase grain yield due to regulation of inflorescence architecture and grain number per spike in wheat and soybean (Wang et al., 2022; Li et al., 2023a). The increase of grain number per spike is often linked with a decrease in grain weight directly influencing TKW.

For PH, EN uniquely identified 14 MTAs; out of this, the following five were associated to the candidate genes S1B_670929735, S3A_646672662, S4A_4593612, S7A_22981667, and S7B_695381826. The first one is located near a cluster of S-type anion channel genes (SLAH2 family: *TraesCS1B02G456200*, *TraesCS1B02G456300*, *TraesCS1B02G456400*, and *TraesCS1B02G456500*), which are associated with nitrate transport (Kumar et al., 2022) and regulation of nitrate flux between root and shoot (Maierhofer et al., 2014), thereby potentially influencing overall plant growth and development, including height. The other marker of relevance is S3A_646672662, which is located near *TraesCS3A02G399800*, a homologue of *GA20ox-D3*, which is catalyzing a late step in gibberellin biosynthesis and is responsible for unidimensional cell growth (Qin et al., 2013). This gene underlies classic “Green Revolution” height phenotypes in rice and wheat (sd1/Rht) and controls stem elongation in legumes (Spielmeyer et al., 2002). The other marker, S4A_4593612, encodes ABC transporter B family member 19, which is well-characterized as a major auxin exporter in *Arabidopsis*. The phytohormone auxin plays a critical role in plant development, in cell growth, division, and expansion (Zhao et al., 2013). This could directly influence the plant height in wheat, making this a promising candidate gene. Furthermore, S7A_22981667 is located close to the gene *GRF5-A1* belonging to the GRFs, which have been mentioned already in the chapter above. And finally, S7B_695381826 is located close to *TraesCS7B02G426100* which is encoding a F-box domain protein, known to be relevant for various functions (Rameneni et al., 2018).

Collectively, these novel ML-derived MTAs enrich our understanding of the genetic architecture of TKW and PH by revealing hormone-related, nutrient-transport, and protein-turnover genes that traditional GWAS alone failed to detect. Especially, penalized models efficiently rank markers and show promise as ML-based GWAS approaches. Moreover, several of the MTAs mapped to genes involved in biotic and abiotic stress responses, pathways we did not explicitly include, but which can profoundly influence resource allocation, developmental timing, and ultimately affecting grain weight (TKW) and stem elongation (PH) under field conditions.

## 5 Conclusion

Machine Learning (ML) offers a powerful complement to traditional GWAS methodologies for plant genomics, as demonstrated in our study using a large-scale, real-world wheat dataset. While mixed linear models remain robust for correcting population structure and controlling false positives, they often miss complex, non-additive genetic signals. ML methods address this limitation by recapitulating key loci detected by conventional approaches, but also by uncovering novel and biologically plausible associations. These findings illustrate how tree-based and penalized regression models can detect even subtle and potentially epistatic effects that evade detection by traditional GWAS tools. Moreover, ML methods offer scalable, interpretable, and model-agnostic importance measures, rendering them especially valuable for high-dimensional plant genomic datasets. By systematically comparing traditional and ML-based GWAS tools, we provide actionable insights into their performance, usability, and biological relevance, advocating for the integration of ML into routine breeding workflows to enhance trait dissection and accelerate marker-assisted selection under complex genomic architectures. Instead of recommending one tool (e.g., the one with the most MTAs) or one method (e.g., the fastest), we have shown that a mix of methods and tools provides a more complete overview, allows a core set of MTAs to be defined, and enables tool/method-specific MTAs to be characterized, which have the potential to yield new insights.

However, several limitations of this study should be considered. First, the phenotypic data come from a large, publicly available breeding dataset and may contain noise from environmental variation, measurement error, and unmodeled genotype-by-environment (G×E) interactions, potentially reducing statistical power to detect MTAs. Second, while ML models capture non-linear effects, they are prone to overfitting in high-dimensional genomic settings. Third, applying and comparing multiple GWAS models introduces an additional layer of multiple testing at the method-comparison level, which may inflate the probability of false-positive findings. To mitigate this, we emphasized overlap across methods and focused on biologically plausible MTAs supported by functional annotation. Our results also differ from Juliana et al. (2019), likely due to methodological differences, including a lenient Bonferroni significance threshold (α = 0.20) and SNP filtering and imputation strategies retaining low-frequency alleles. Nevertheless, we replicated five loci reported by Juliana et al. (2019): three for PH (S3B_757480752 via BLINK, FarmCPU, RF, XGB, S6A_484916096 via BLINK only, and S7A_403940100 via BLINK, FarmCPU, MLMM, sommer, Tassel, RF), and one for TKW (S6B_583334052 via FarmCPU), and one locus that they associated with TKW, but we found a significant association for PH (S6A_553941375 via BLINK). In contrast, we did not replicate MTAs at the GS5-3A locus, which has been linked to increased kernel size (Ma et al., 2016), or the *Tasus* gene on chromosome 7B.

To fully harness ML’s potential for GWAS, future research must integrate technical innovation with domain expertise. Hybrid analytical frameworks that incorporate interaction effects and biological priors, such as gene regulatory networks, functional ontologies, or chromatin accessibility data, could improve model interpretability and guide learning. Ensemble strategies combining traditional statistical GWAS, ML, and causal inference may further disentangle signals from noise, addressing missing heritability and extending applicability to complex dynamic traits and G×E interactions through multi-environmental and longitudinal phenotyping. Furthermore, follow-up analysis of the top consensus markers and interesting ML-derived MTAs, including investigation of potential interaction effects, SHAP-based estimation of model-specific effect sizes, and subsequent fine-mapping and haplotype-level characterization, will be essential to validate these loci and to distinguish true causal variants from markers identified through correlation or linkage alone. Likewise, extending ML-GWAS to explicitly capture genotype-by-environment interactions will enable the identification of environment-specific and stable alleles, thereby strengthening selection decisions and supporting the development of resilient cultivars across variable production environments.

## Data Availability

Publicly available datasets were analyzed in this study. This data can be found here: https://www.nature.com/articles/s41588-019-0496-6.

## Glossary

BLINK: Bayesian information and linkage disequilibrium iteratively nested keyway
CMLM: Compressed mixed linear model
EN: Elastic net
FarmCPU: Fixed and random model circulating probability unification
FDR: False discovery rate
GLM: Generalized linear model
GLMnet: Generalized linear model with elastic net regularization
GWAS: Genome-wide association study
LARS: Least angle regression
LD: Linkage disequilibrium
MAS: Marker-assisted selection
ML: Machine learning
MLM: Mixed linear model
MLMM: Multi-locus mixed linear model
MTA: Marker-trait association
PC: Principal component
PH: Plant height
QTL: Quantitative trait locus/loci
QTN: Quantitative trait nucleotide
RF: Random Forest
RMSE: Root mean square error
SUPER: Settlement of MLM under progressively exclusive relationship
SVM: Support vector machine
TKW: Thousand kernel weight
TSLRF: Two Stage algorithm based on Least angle regression and Random Forest
XGB: Extreme gradient boosting
