# Supplementary material for: Applying traditional and machine learning-based GWAS approaches for marker-trait identification in wheat
Source: Front Plant Sci. 2026 Jan 28;16:1734247. doi: 10.3389/fpls.2025.1734247 (PMC12891156; doi:10.3389/fpls.2025.1734247)
Supplement: Supplementary file 1 [file Presentation1.pptx]

## Slide 1
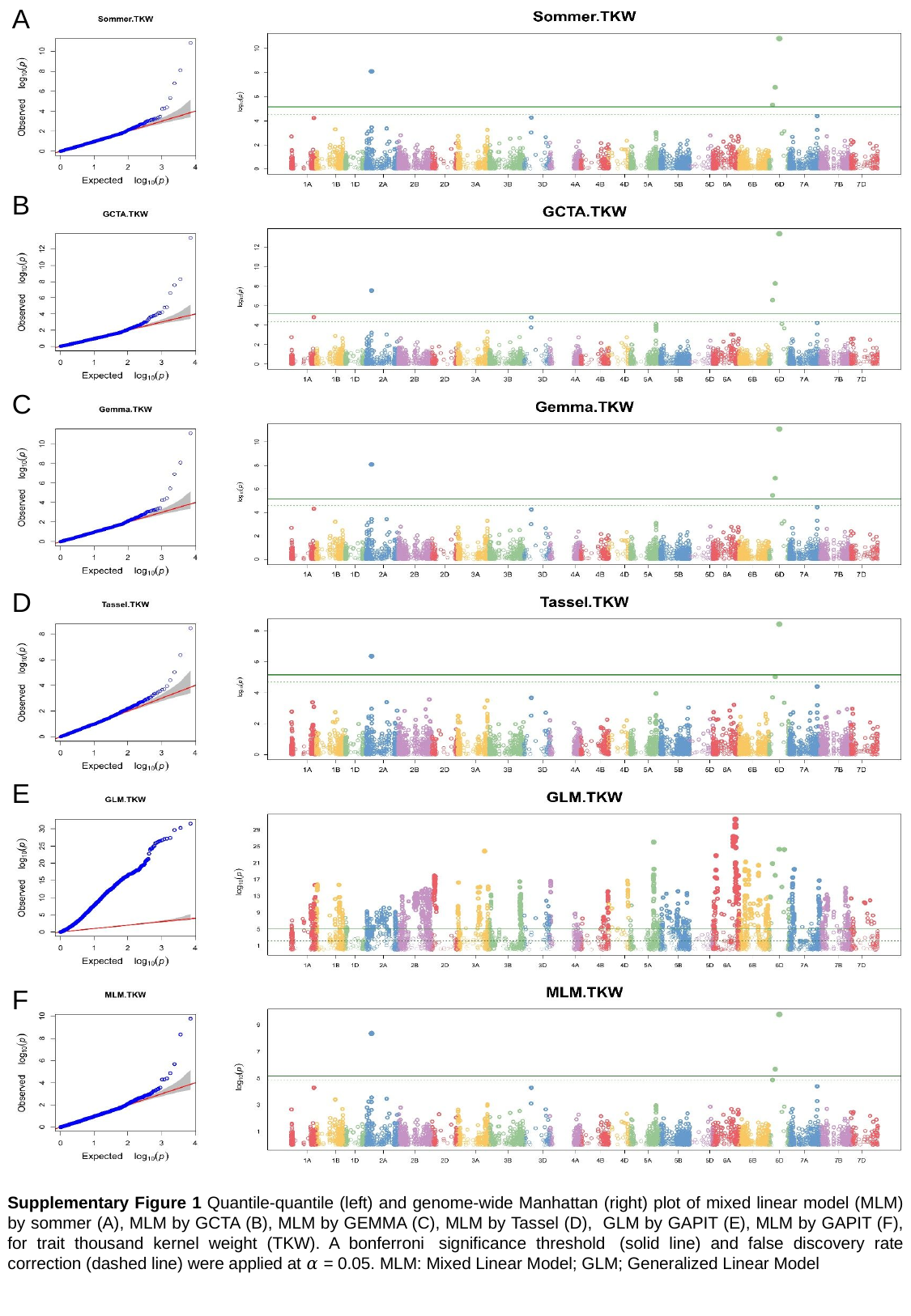

A
B
C
D
E
F
Supplementary Figure 1 Quantile-quantile (left) and genome-wide Manhattan (right) plot of mixed linear model (MLM) by sommer (A), MLM by GCTA (B), MLM by GEMMA (C), MLM by Tassel (D), GLM by GAPIT (E), MLM by GAPIT (F), for trait thousand kernel weight (TKW). A bonferroni  significance threshold  (solid line) and false discovery rate correction (dashed line) were applied at 𝛼 = 0.05. MLM: Mixed Linear Model; GLM; Generalized Linear Model

## Slide 2
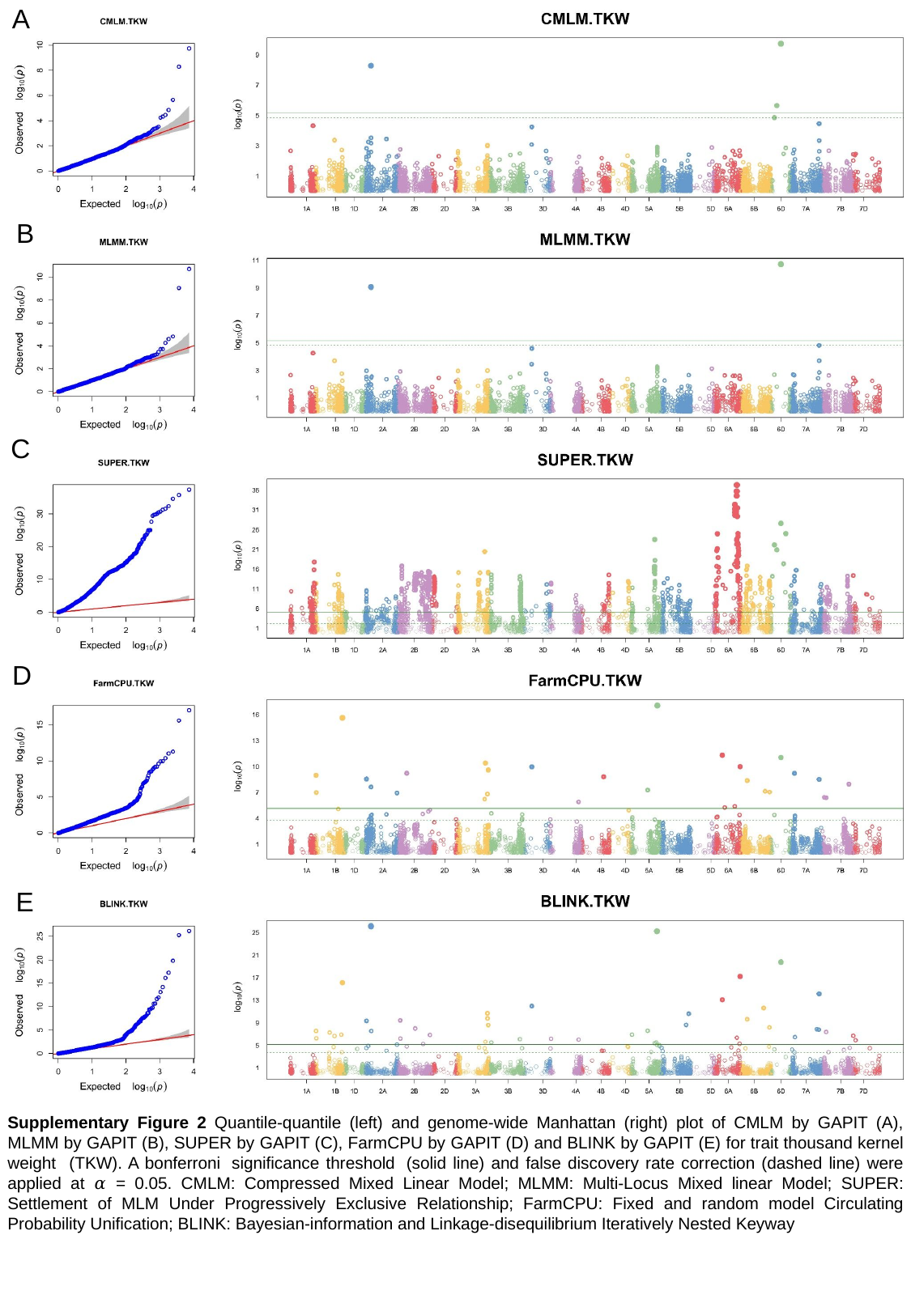

A
B
C
D
E
Supplementary Figure 2 Quantile-quantile (left) and genome-wide Manhattan (right) plot of CMLM by GAPIT (A), MLMM by GAPIT (B), SUPER by GAPIT (C), FarmCPU by GAPIT (D) and BLINK by GAPIT (E) for trait thousand kernel weight (TKW). A bonferroni  significance threshold  (solid line) and false discovery rate correction (dashed line) were applied at 𝛼 = 0.05. CMLM: Compressed Mixed Linear Model; MLMM: Multi-Locus Mixed linear Model; SUPER: Settlement of MLM Under Progressively Exclusive Relationship; FarmCPU: Fixed and random model Circulating Probability Unification; BLINK: Bayesian-information and Linkage-disequilibrium Iteratively Nested Keyway

## Slide 3
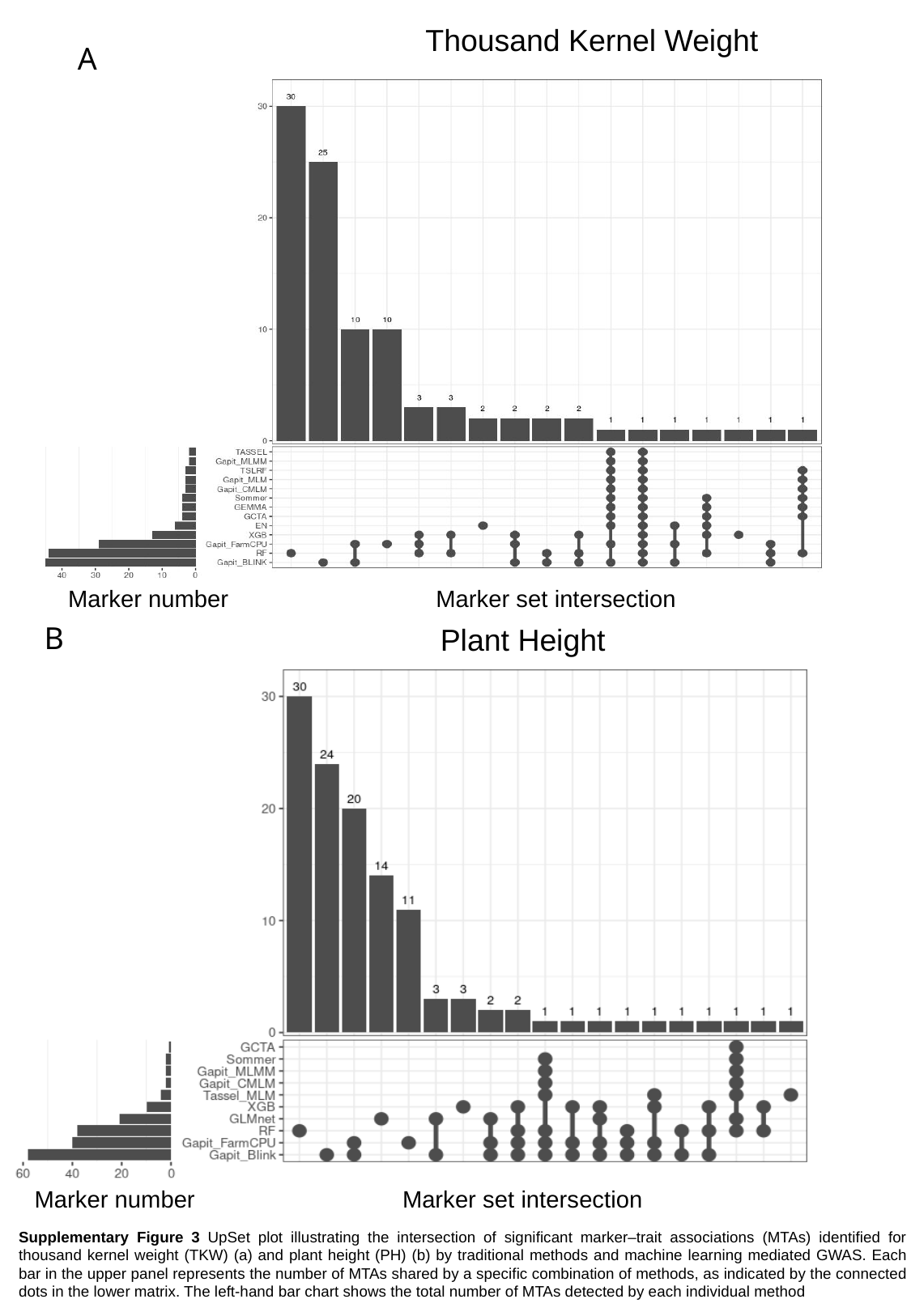

Thousand Kernel Weight
A
Marker number
Marker set intersection
B
Plant Height
Marker number
Marker set intersection
Supplementary Figure 3 UpSet plot illustrating the intersection of significant marker–trait associations (MTAs) identified for thousand kernel weight (TKW) (a) and plant height (PH) (b) by traditional methods and machine learning mediated GWAS. Each bar in the upper panel represents the number of MTAs shared by a specific combination of methods, as indicated by the connected dots in the lower matrix. The left-hand bar chart shows the total number of MTAs detected by each individual method

## Slide 4
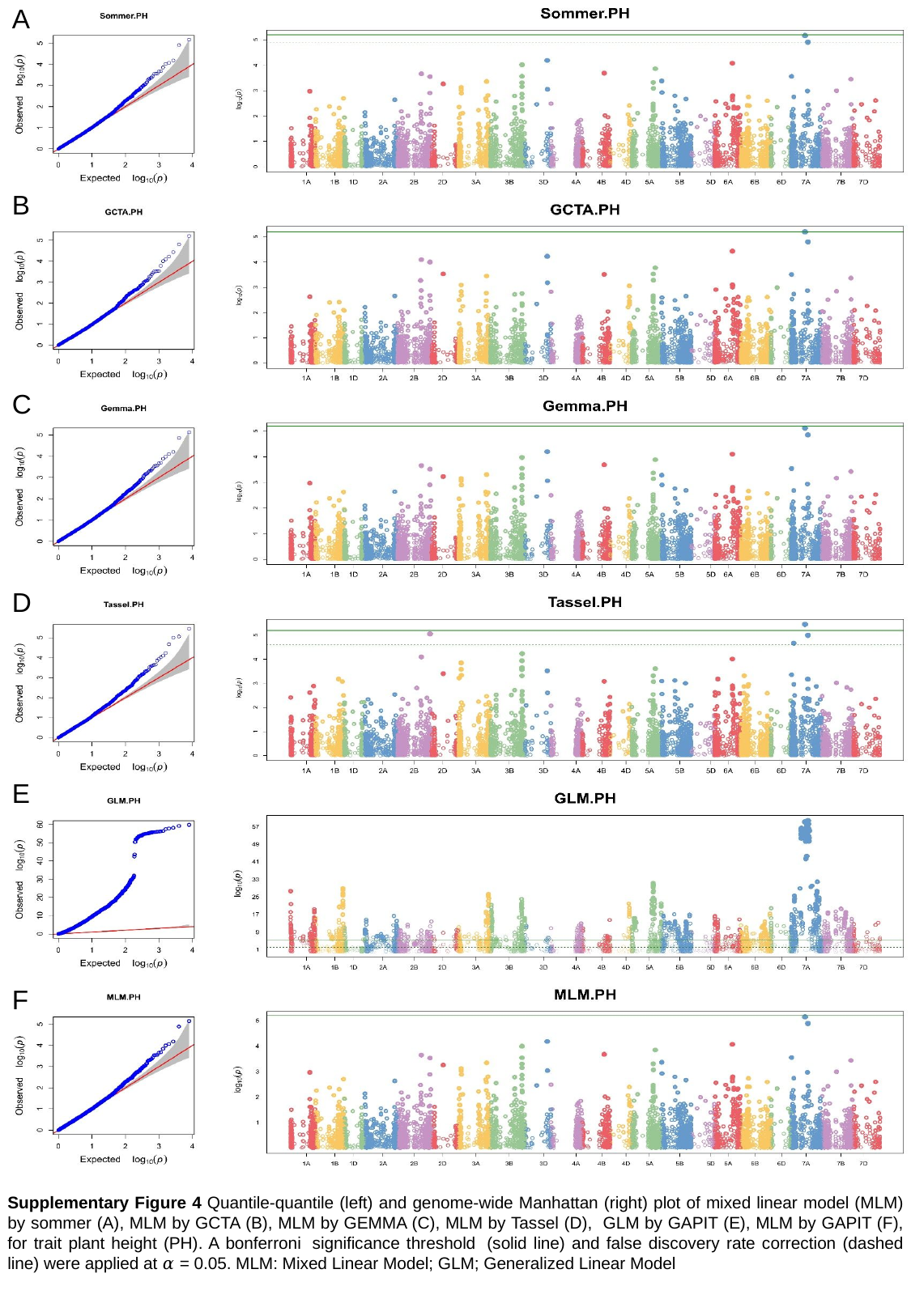

A
B
C
D
E
F
Supplementary Figure 4 Quantile-quantile (left) and genome-wide Manhattan (right) plot of mixed linear model (MLM) by sommer (A), MLM by GCTA (B), MLM by GEMMA (C), MLM by Tassel (D), GLM by GAPIT (E), MLM by GAPIT (F), for trait plant height (PH). A bonferroni  significance threshold  (solid line) and false discovery rate correction (dashed line) were applied at 𝛼 = 0.05. MLM: Mixed Linear Model; GLM; Generalized Linear Model

## Slide 5
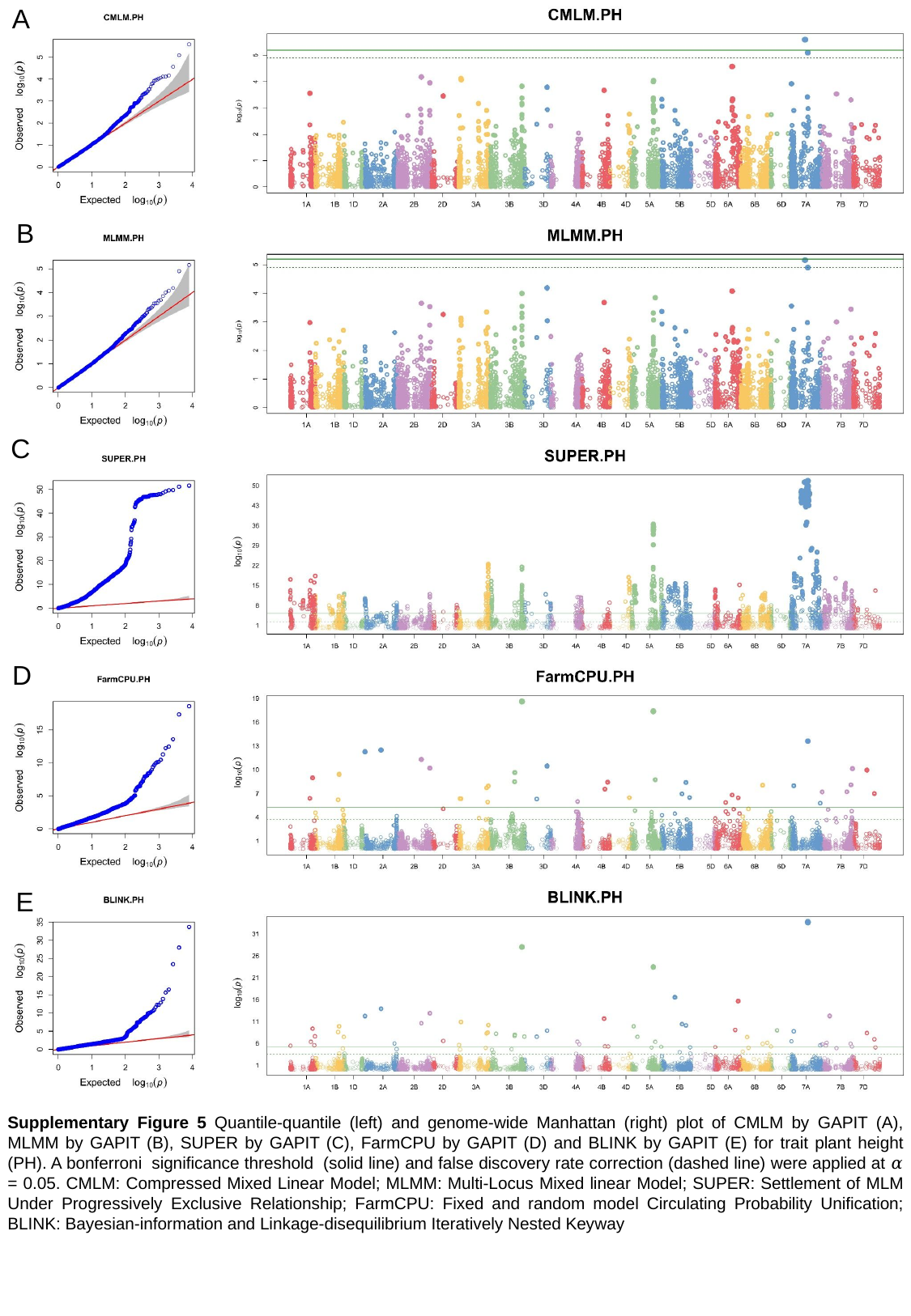

A
B
C
D
E
Supplementary Figure 5 Quantile-quantile (left) and genome-wide Manhattan (right) plot of CMLM by GAPIT (A), MLMM by GAPIT (B), SUPER by GAPIT (C), FarmCPU by GAPIT (D) and BLINK by GAPIT (E) for trait plant height (PH). A bonferroni  significance threshold  (solid line) and false discovery rate correction (dashed line) were applied at 𝛼 = 0.05. CMLM: Compressed Mixed Linear Model; MLMM: Multi-Locus Mixed linear Model; SUPER: Settlement of MLM Under Progressively Exclusive Relationship; FarmCPU: Fixed and random model Circulating Probability Unification; BLINK: Bayesian-information and Linkage-disequilibrium Iteratively Nested Keyway

## Slide 6
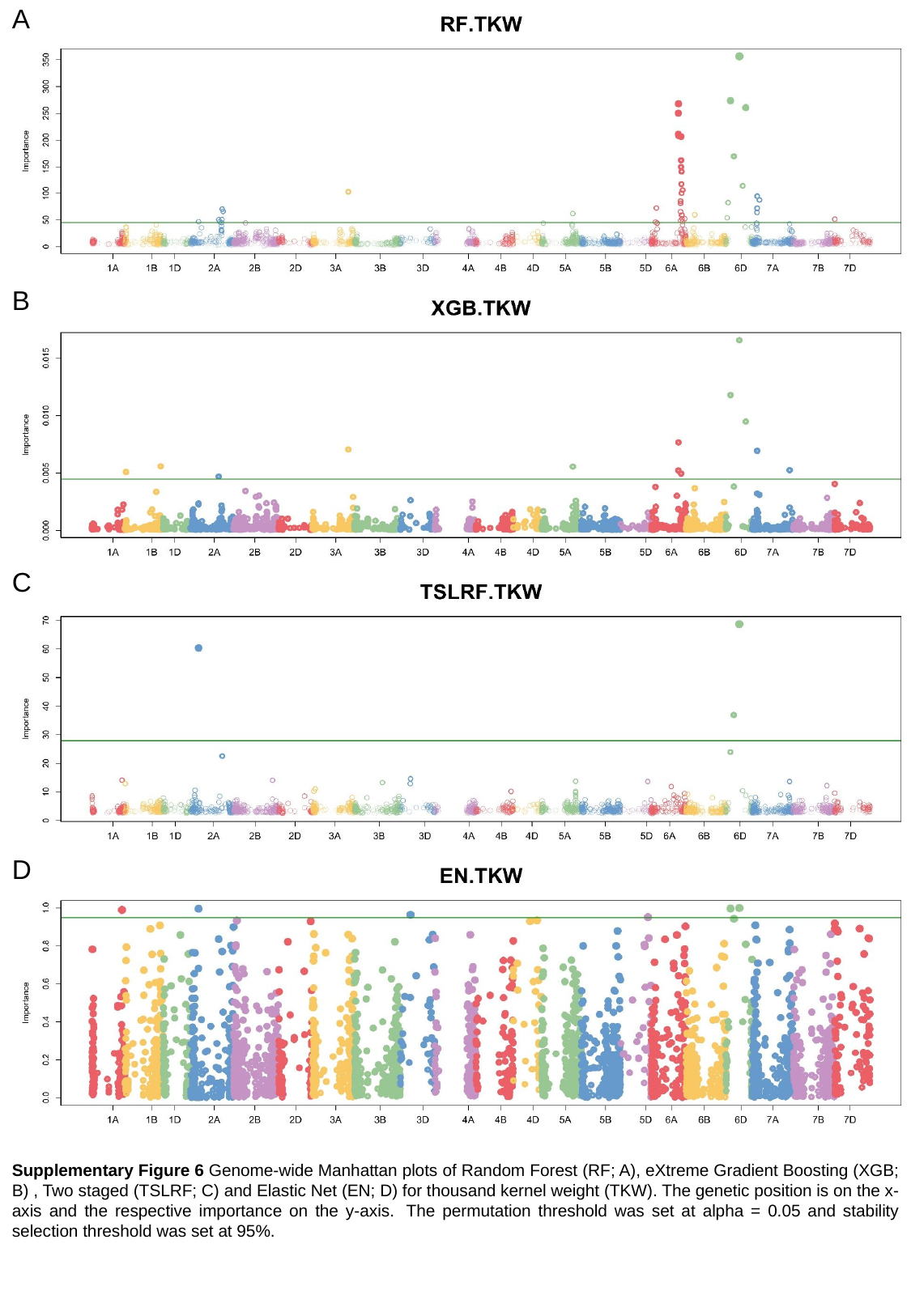

A
B
C
D
Supplementary Figure 6 Genome-wide Manhattan plots of Random Forest (RF; A), eXtreme Gradient Boosting (XGB; B) , Two staged (TSLRF; C) and Elastic Net (EN; D) for thousand kernel weight (TKW). The genetic position is on the x-axis and the respective importance on the y-axis.  The permutation threshold was set at alpha = 0.05 and stability selection threshold was set at 95%.

## Slide 7
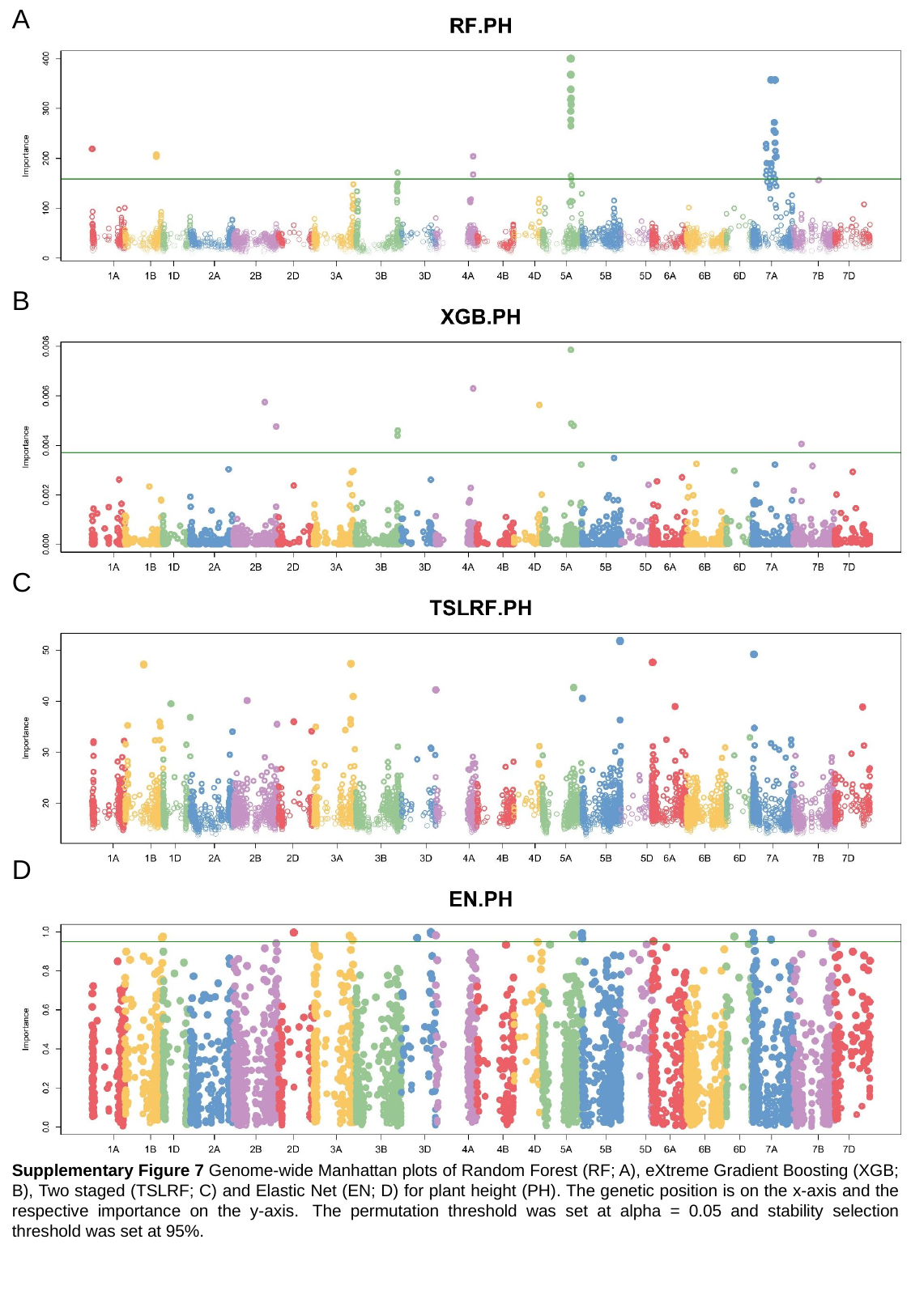

A
B
C
D
Supplementary Figure 7 Genome-wide Manhattan plots of Random Forest (RF; A), eXtreme Gradient Boosting (XGB; B), Two staged (TSLRF; C) and Elastic Net (EN; D) for plant height (PH). The genetic position is on the x-axis and the respective importance on the y-axis.  The permutation threshold was set at alpha = 0.05 and stability selection threshold was set at 95%.
