# Supplementary material for: Applying traditional and machine learning-based GWAS approaches for marker-trait identification in wheat
Source: Front Plant Sci. 2026 Jan 28;16:1734247. doi: 10.3389/fpls.2025.1734247 (PMC12891156; doi:10.3389/fpls.2025.1734247)
Supplement: Supplementary file 2 [file Presentation2.pptx]

## Slide 1
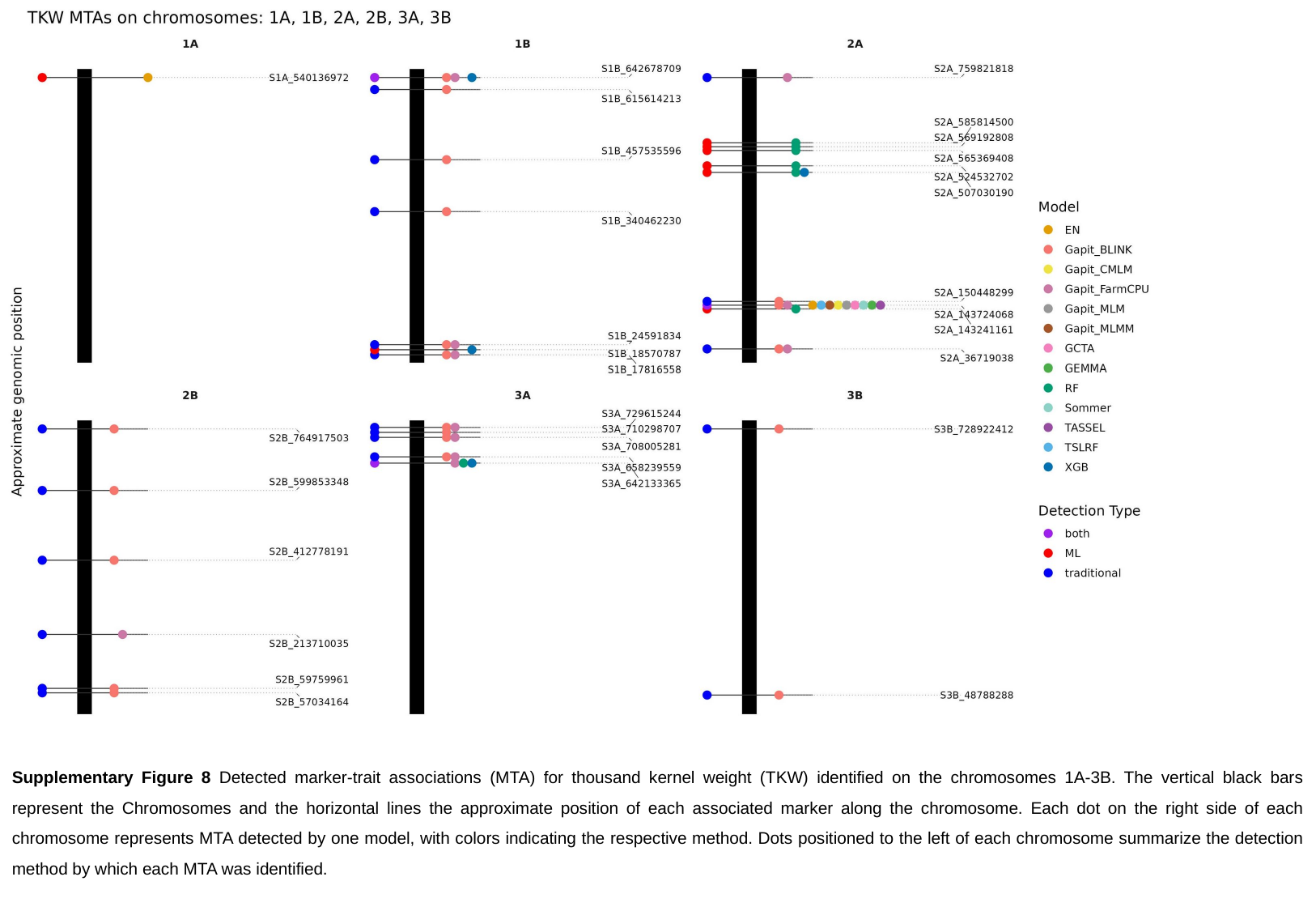

Supplementary Figure 8 Detected marker-trait associations (MTA) for thousand kernel weight (TKW) identified on the chromosomes 1A-3B. The vertical black bars represent the Chromosomes and the horizontal lines the approximate position of each associated marker along the chromosome. Each dot on the right side of each chromosome represents MTA detected by one model, with colors indicating the respective method. Dots positioned to the left of each chromosome summarize the detection method by which each MTA was identified.

## Slide 2
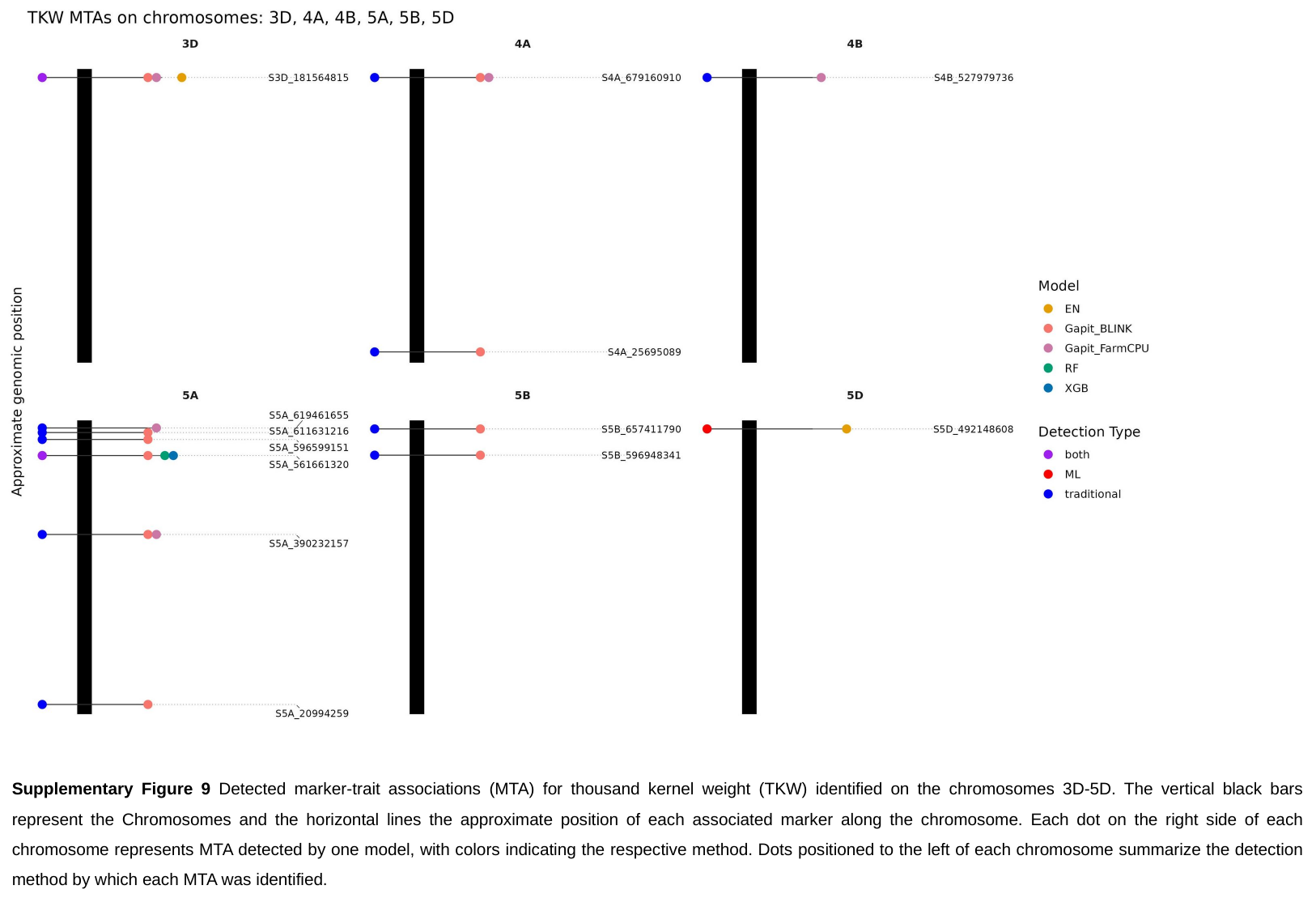

Supplementary Figure 9 Detected marker-trait associations (MTA) for thousand kernel weight (TKW) identified on the chromosomes 3D-5D. The vertical black bars represent the Chromosomes and the horizontal lines the approximate position of each associated marker along the chromosome. Each dot on the right side of each chromosome represents MTA detected by one model, with colors indicating the respective method. Dots positioned to the left of each chromosome summarize the detection method by which each MTA was identified.

## Slide 3
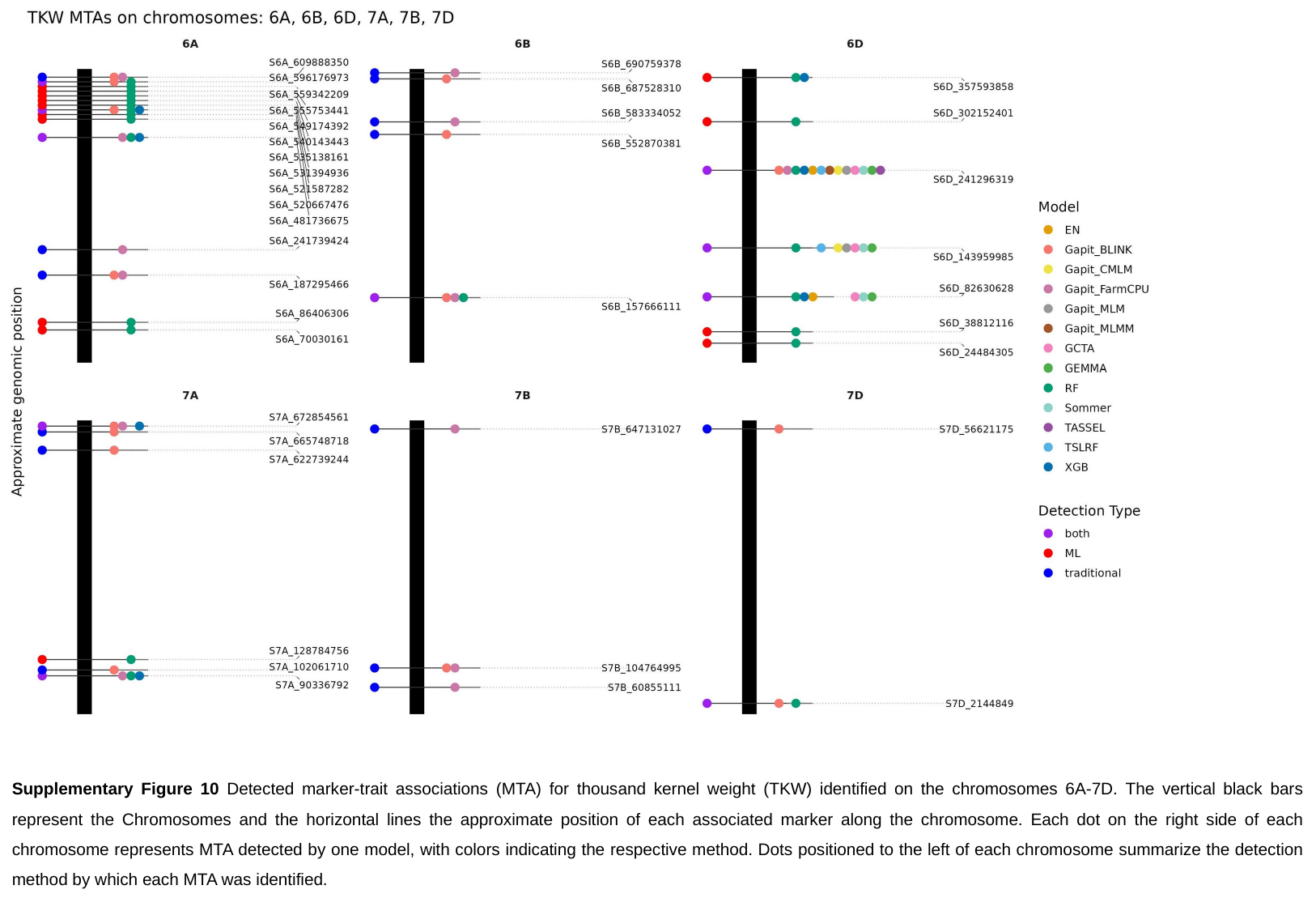

Supplementary Figure 10 Detected marker-trait associations (MTA) for thousand kernel weight (TKW) identified on the chromosomes 6A-7D. The vertical black bars represent the Chromosomes and the horizontal lines the approximate position of each associated marker along the chromosome. Each dot on the right side of each chromosome represents MTA detected by one model, with colors indicating the respective method. Dots positioned to the left of each chromosome summarize the detection method by which each MTA was identified.

## Slide 4
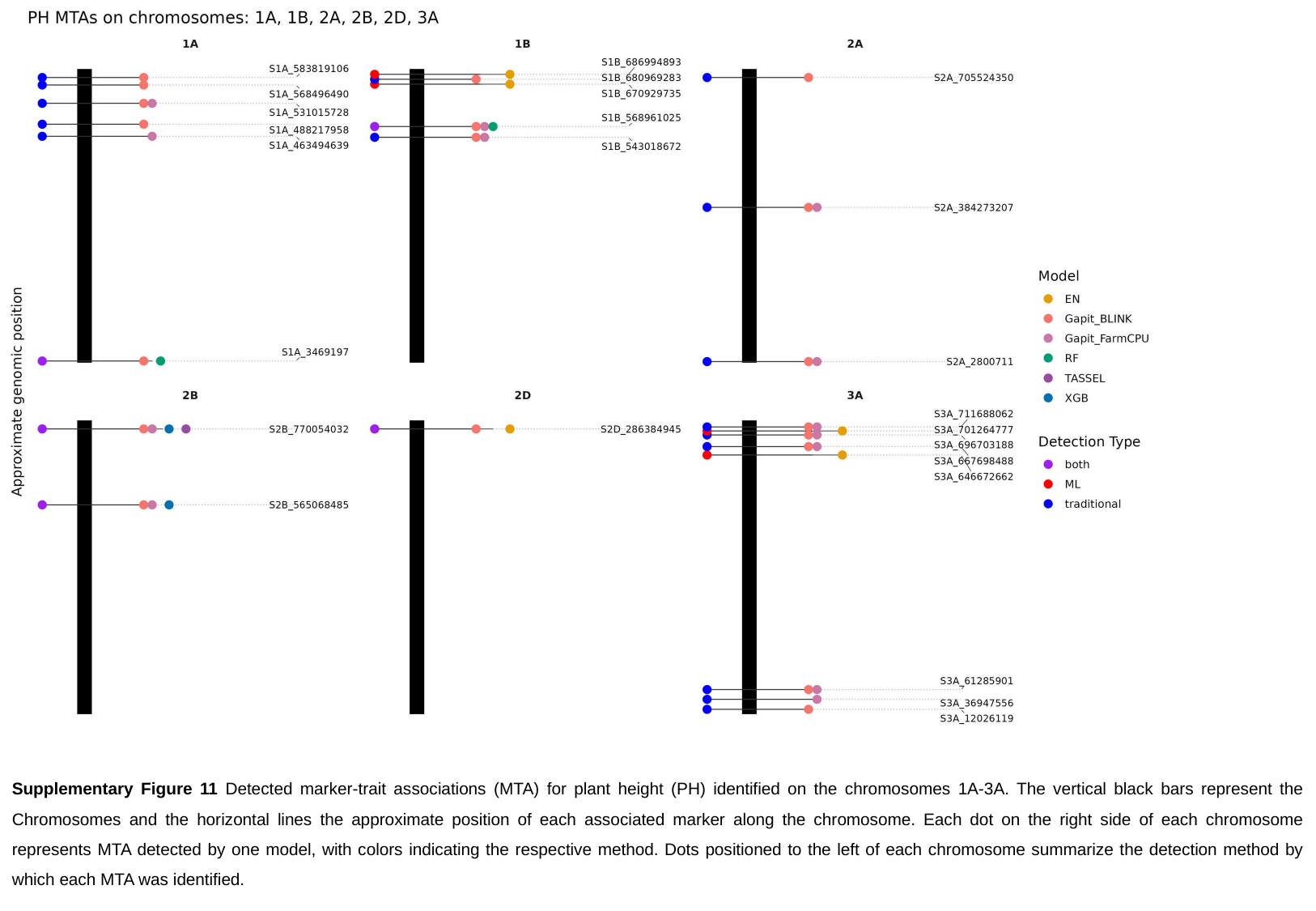

Supplementary Figure 11 Detected marker-trait associations (MTA) for plant height (PH) identified on the chromosomes 1A-3A. The vertical black bars represent the Chromosomes and the horizontal lines the approximate position of each associated marker along the chromosome. Each dot on the right side of each chromosome represents MTA detected by one model, with colors indicating the respective method. Dots positioned to the left of each chromosome summarize the detection method by which each MTA was identified.

## Slide 5
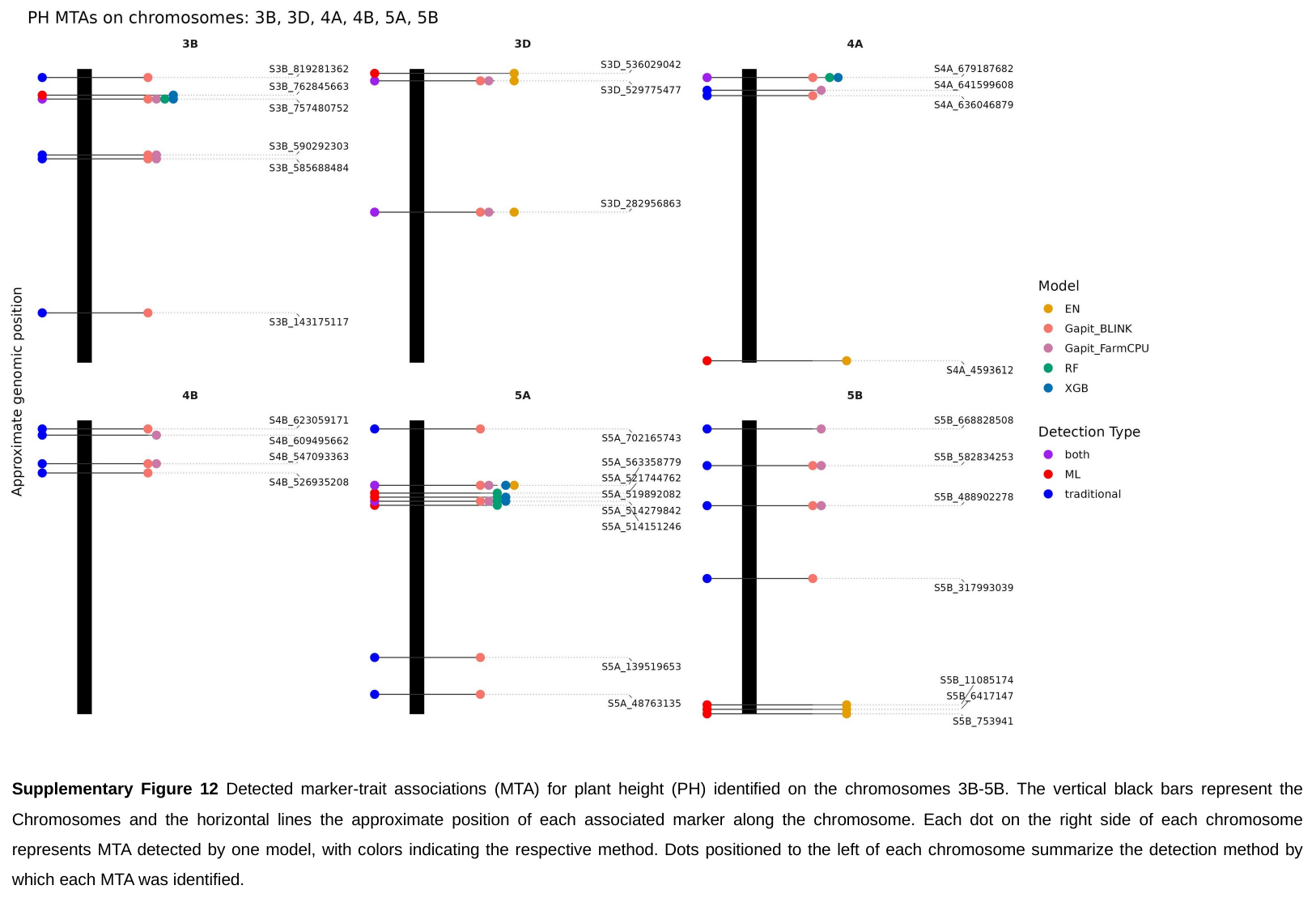

Supplementary Figure 12 Detected marker-trait associations (MTA) for plant height (PH) identified on the chromosomes 3B-5B. The vertical black bars represent the Chromosomes and the horizontal lines the approximate position of each associated marker along the chromosome. Each dot on the right side of each chromosome represents MTA detected by one model, with colors indicating the respective method. Dots positioned to the left of each chromosome summarize the detection method by which each MTA was identified.

## Slide 6
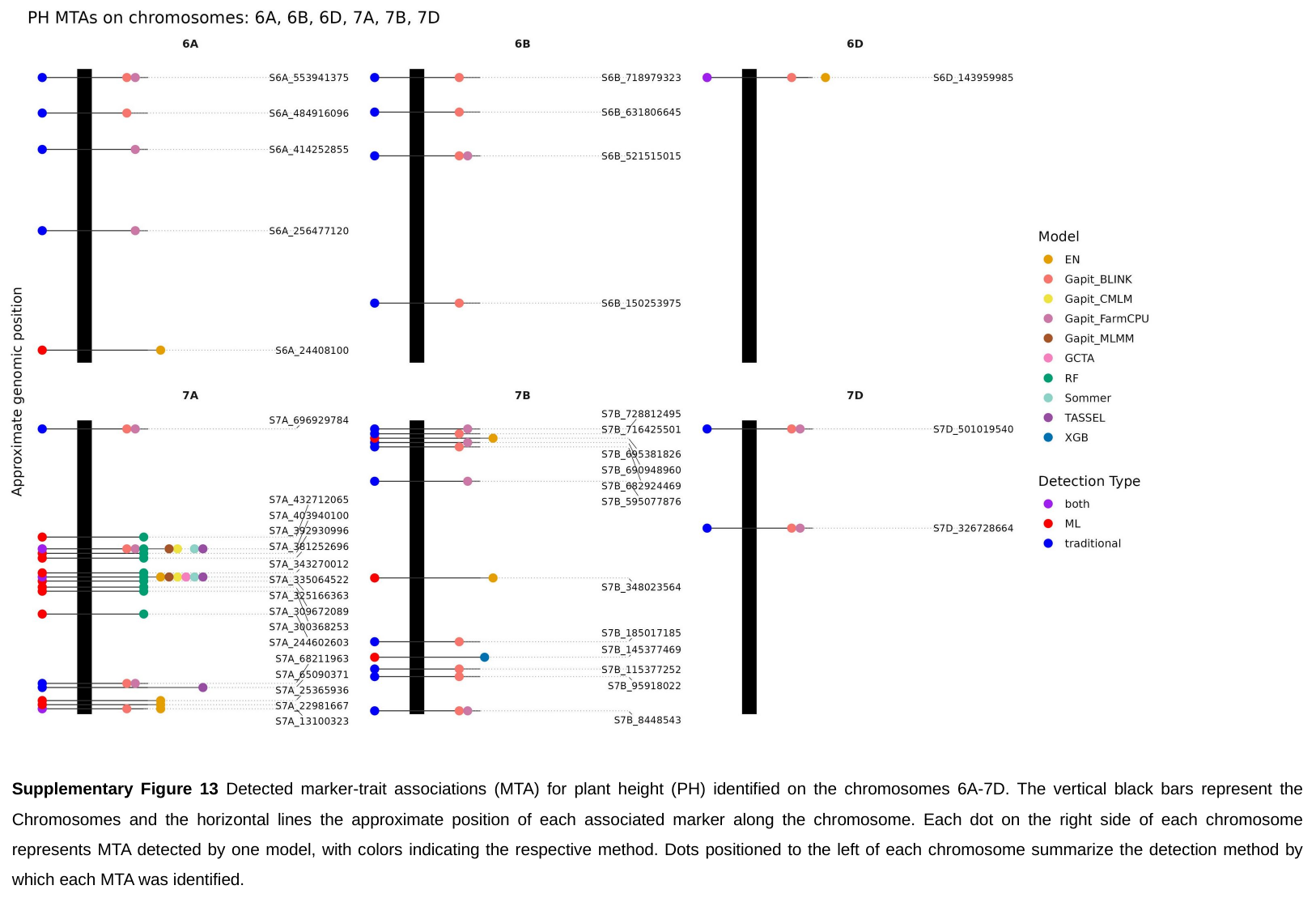

Supplementary Figure 13 Detected marker-trait associations (MTA) for plant height (PH) identified on the chromosomes 6A-7D. The vertical black bars represent the Chromosomes and the horizontal lines the approximate position of each associated marker along the chromosome. Each dot on the right side of each chromosome represents MTA detected by one model, with colors indicating the respective method. Dots positioned to the left of each chromosome summarize the detection method by which each MTA was identified.

## Slide 7
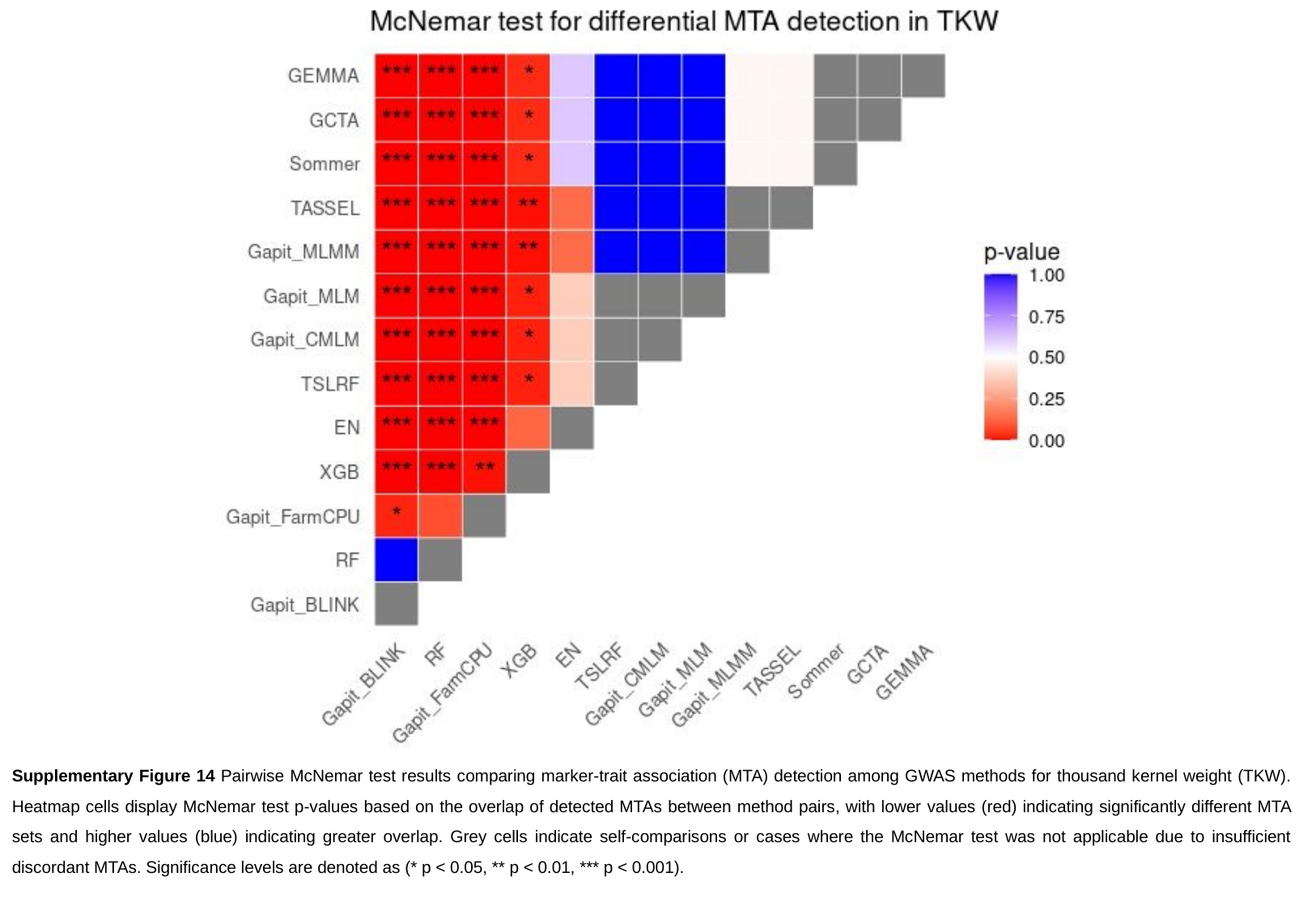

Supplementary Figure 14 Pairwise McNemar test results comparing marker-trait association (MTA) detection among GWAS methods for thousand kernel weight (TKW). Heatmap cells display McNemar test p-values based on the overlap of detected MTAs between method pairs, with lower values (red) indicating significantly different MTA sets and higher values (blue) indicating greater overlap. Grey cells indicate self-comparisons or cases where the McNemar test was not applicable due to insufficient discordant MTAs. Significance levels are denoted as (* p < 0.05, ** p < 0.01, *** p < 0.001).

## Slide 8
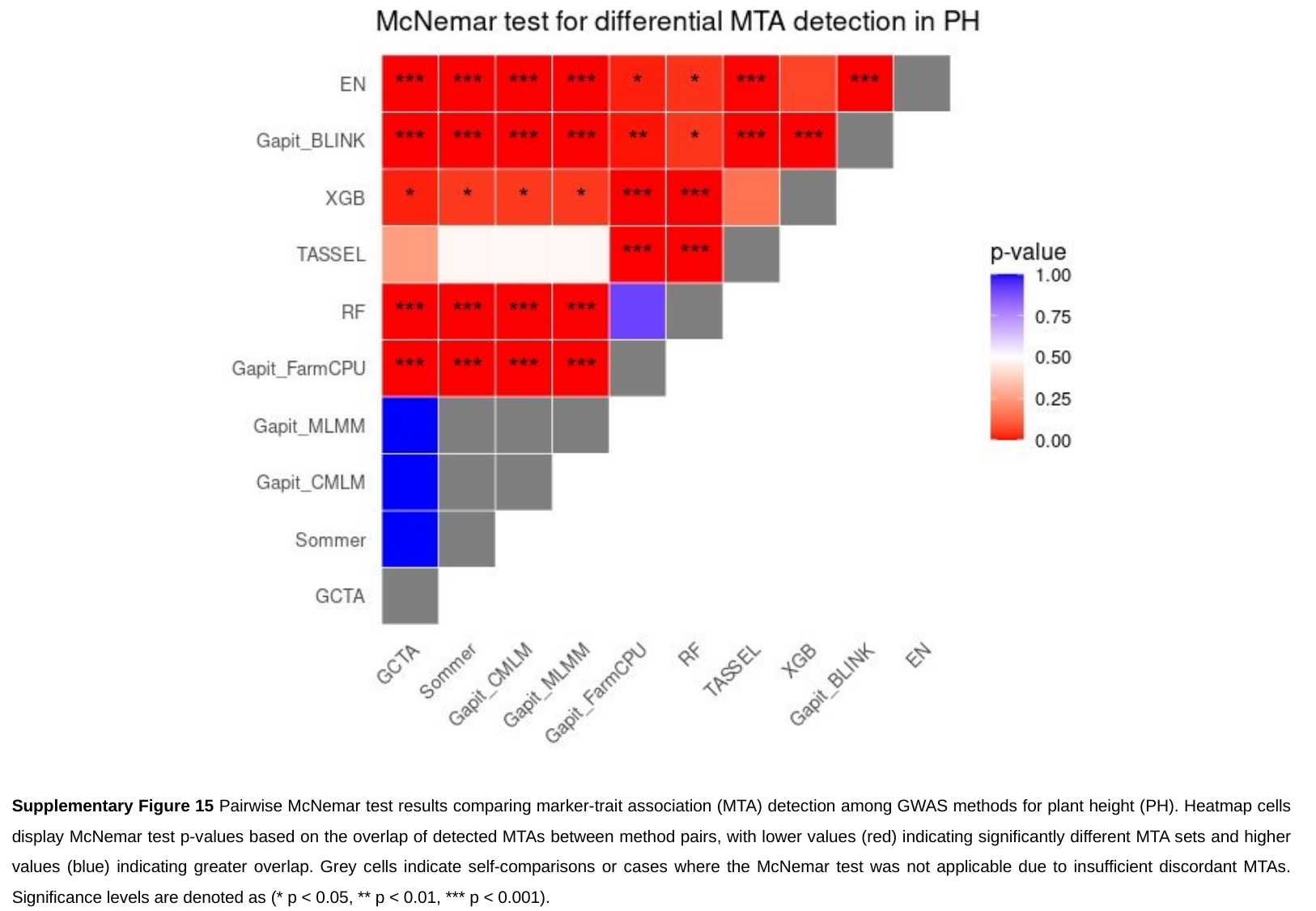

Supplementary Figure 15 Pairwise McNemar test results comparing marker-trait association (MTA) detection among GWAS methods for plant height (PH). Heatmap cells display McNemar test p-values based on the overlap of detected MTAs between method pairs, with lower values (red) indicating significantly different MTA sets and higher values (blue) indicating greater overlap. Grey cells indicate self-comparisons or cases where the McNemar test was not applicable due to insufficient discordant MTAs. Significance levels are denoted as (* p < 0.05, ** p < 0.01, *** p < 0.001).
